# Numerical investigation on the torsional improvement of reinforced concrete beams strengthened with various techniques

**DOI:** 10.1038/s41598-026-38794-z

**Published:** 2026-03-10

**Authors:** M. A. Yusuf, M. S. Zahran, A. Osman, Nabil. M. Nagy

**Affiliations:** Department of Civil Engineering, Military Technical College, Cairo, Egypt

**Keywords:** Torsion, Strengthening, RC beams, Steel mesh, FRP, GFRP, Engineering, Materials science

## Abstract

This study digitally investigates the torsional behavior of reinforced concrete (RC) beams strengthened with near-surface bracing (NSM) and external bracing using Abaqus/CAE software. Finite element analysis (FE) was developed based on a previously validated experimental program, encompassing five tested beams, thus providing a realistic basis for model validation. The numerical results showed strong agreement with experimental trends, with deviations of less than 5%, confirming the model’s accuracy and reliability. The analysis utilized the concrete deterioration plasticity (CDP) model, realistic surface bonding properties, and the elastic steel behavior to effectively monitor cracking and stiffness degradation. The grid sensitivity indicated that a 25 × 25 mm element size achieved optimal accuracy and efficiency, while an extension angle of ψ = 37° best represented the torsional response. The results showed that overlap lengths between 0.6d and 0.8d in the NSM stirrups enhanced torsional strength and elasticity, achieving a 110–138% increase in ultimate moment and a 14–86% increase in torsional angle compared to the control beam. Furthermore, combining the NSM stirrups with externally bonded steel mesh layers improved torsional performance up to three layers, after which the improvement stabilized. The developed finite element (FE) model proved to be a reliable and practical tool for analyzing, predicting, and optimizing torsional reinforcement systems in reinforced concrete beams. The study also investigated the effect of inclined bracing on beam faces compared to vertical bracing, demonstrating that inclined bracing exhibited a very high efficiency in resisting torsional stress, reaching 338%, a significantly higher percentage compared to vertical bracing, thus confirming its effectiveness.

## Introduction

Reinforced concrete (RC) structures are typically subjected to various loading conditions, often involving significant torsional moments. These torsional effects can have a profound impact on the structural response and overall stability of members, particularly cable-stayed beams, ring beams, and many bridge girders and end beams. In many practical applications, it is necessary to reinforce RC members that exhibit insufficient torsional shear capacity to ensure safety and serviceability. The need for torsional reinforcement may arise for various reasons, including insufficient transverse reinforcement resulting from poor construction practices; material deterioration leading to corrosion and loss of effective reinforcement area; or increased load requirements resulting from changes in occupancy, use, or design modifications over time. Therefore, it is necessary to consider external strengthening methods to resist torsional loads in existing structures. The following is a review of some of the research that has addressed this issue^1,2^.

Many studies have discussed the reinforcement system based on the near surface mounted (NSM) technique, and its concept is based on making cavities on the surface of the concrete beam and placing reinforcing steel, fiber, or steel ropes and covering them with an adhesive material^3–5^.

This technique had an effective impact in strengthening concrete beams exposed to twisting, as a comparison was made in many studies between the reference beam that was not reinforced and the beams reinforced with this technique, and the large difference between the reinforced beams and the reference beam was made clear, which may indicate the success of this technique^6–9^.

Patane, et al. conducted an experimental and numerical study to evaluate the effect of reinforcing reinforced concrete beams with BFRP fabrics on their torsional resistance. The study included four beams with dimensions of 150 × 250 × 2100 mm, one of which was unreinforced and three reinforced with different BFRP laminate configurations (full U-twist, 150- and 300-mm strip twists at 150 mm spacing). The beams were subjected to a pure torsional test, and the results were analyzed using ANSYS after modeling in SOLIDWORKS. The results showed that full U-twist reinforcement was the most effective in improving the torsional resistance^10^.

Franco et al. conducted a study to evaluate the efficiency of stainless-steel reinforcement in strengthening the flexural strength of reinforced concrete T-beams using EBR, NSM, and MA-EBR bonding techniques through experimental tests and finite element (FE) analysis. An experimental test was conducted to obtain realistic results, which helped improve the accuracy of the numerical modeling and predict the behavior of the beams up to rupture. The results showed that all strengthening techniques improved the flexural stiffness, but EBR and NSM beams suffered premature rupture, while the MA-EBR technique performed best in terms of ductility and load-bearing capacity, making it the most efficient technique^11,12^.

Hamoda et al. presented a comprehensive experimental and numerical investigation into the torsional and shear strengthening of reinforced concrete beams using ferrocement techniques, glass fiber-reinforced polymer (GFRP) materials, and various reinforcing meshes. The experiments included slotted and deep-slotted beams subjected to torsion and shear tests, with three-dimensional nonlinear analysis using ABAQUS and ANSYS to numerically verify the structural behavior up to failure. The results showed that fully encasing GFRP or ferrocement increased the ultimate torsional moment by 40.63% to 81.25% compared to unreinforced beams, and that using expanded metal mesh instead of welded wire mesh improved the torsional strength by 9.43%. Multiple layers of ferrocement increased the strength by up to 75%, while fasteners increased the performance by 9.1%. In deep beams, SHCC mesh casings increased the ultimate load by 82%, the cracking load by 73%, the elastic stiffness by 457%, and the energy absorption by 380%. Numerical modeling demonstrated high accuracy in representing failure modes and buckling angles, confirming that mesh-reinforced ferrocement is an effective and economical solution for strengthening concrete beams compared to EB-GFRP techniques^13–15^.

Alabdulhady et al. studied the behavior of reinforced concrete beams under torsional and combined loads, evaluating external reinforcement techniques using carbon fiber-reinforced polymer (CFRP), aramid fibers, and EB-FRP/FRCM systems. The results show that external reinforcement improves flexural strength, but its effectiveness is affected by the interaction of torsional, flexural, and shear forces, coil configuration, and fiber type. Tests and numerical modeling also demonstrated that aramid fibers reduce brittle fracture and increase flexural capacity, while EB-FRP/FRCM systems proved highly effective in improving the flexural performance of reinforced concrete beams^16–18^.

This study investigates the effect of inclined rectangular helical stirrups on improving the torsional behavior of solid and hollow-core reinforced concrete beams. The experimental program included ten beams of varying reinforcement, with three-dimensional numerical analysis using ANSYS 15.0 to verify the results. The results showed that the use of inclined helical stirrups enhanced the torsional capacity by 16% and 18% for the solid and hollow beams, respectively, and increased the strain energy by approximately 27% and 16%, with more flexible bending compared to conventional stirrups. The numerical model also showed good agreement with the experimental results, confirming the effectiveness of this technique in improving torsional performance^19^.

Previously, numerous studies have discussed the effect of different strengthening techniques on beams under torsion. Experiments demonstrated realistic results, and some beams were modeled using finite element software. However, there was a significant gap in the lack of comprehensive research to compare and contrast strengthening methods, to better understand their effectiveness, and to examine the advantages in terms of implementation and ease of use.

Most previous studies have concentrated on a single torsional strengthening technique such as NSM, EBR, or FRP systems without providing a unified and systematic numerical comparison among different strengthening systems under pure torsional loading. In addition, many numerical investigations have been limited to validating a single model, without performing a comprehensive and structured parametric analysis that considers key influencing parameters, including the overlap length of external stirrups, the number of external mesh layers, and the interaction between NSM and EBR techniques. Furthermore, a clear research gap remains in the lack of direct linkage between experimentally validated numerical results and the optimization of strengthening configurations in terms of both mechanical performance and economic efficiency, which restricts the practical applicability and generalization of previous findings.

The novelty and scientific contribution of this research lies in the provision of a unified and accurate numerical framework that enables a systematic comparison between vertical, inclined, and hybrid torsional bracing systems for reinforced concrete beams subjected to pure torsion. The study identifies the optimal ranges for the overlap length of external bracing elements using NSM technology, elucidates the saturation threshold associated with increasing the number of outer mesh layers, and demonstrates the superior mechanical efficiency of inclined bracing systems compared to conventional vertical configurations. Taken together, these findings help bridge the gap between advanced numerical modeling and practical design decisions, providing clear guidance for efficient and economical torsional bracing of reinforced concrete beams.

## Experimental program

### General description

In this study, Abaqus numerical simulation of laboratory beams was used in the experimental program. This experimental simulation aimed to use this beam to develop a validated numerical model. After verifying the success of the experimental model with experimental results, the parametric study was successfully completed. These studies thus examine various parameters not covered in the experimental program, encouraging comprehensive collaboration on the overall performance of beams.

This numerical model is based on the experimental study titled "Experimental Investigation of R.C. Beams Using a New External Strengthening System Under Torsion Load," by Yusuf, et al.^20^, which aimed to improve the torsional performance of reinforced concrete beams using a new external strengthening system based on the Near Surface Mounted (NSM) bar technique.

### Experimental design

**Fig. 1:**
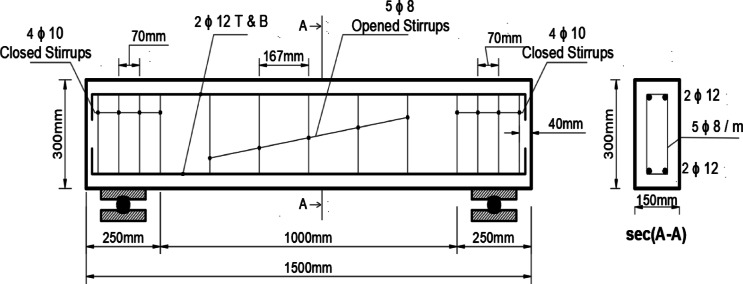
Reinforcement Beams Details^20^.

The beams are designed in accordance with the provisions of ACI 318 code^21^. All beams had a rectangular cross-section with the same nominal dimensions (b = 150 mm wide, d = 300 mm deep, and L = 1500 mm internal reinforcement). Figure 1 shows the dimensions and reinforcement details of the reinforced concrete beams. The beams were subjected to a test area, the NSM bracing system was applied, and the steel torsional internal reinforcement in the transverse direction was calculated according to ACI 318 code. The corresponding reinforcement ratios for the longitudinal and transverse internal reinforcement were ρ_sl =_ (Asl /Ac) _=_ 1.01% and ρ_st =_ {(A_st_ /A_c_) /(p_t_ /s_t_)}_=_ 0.47%, respectively, where (A_sl_) is the total area of ​​the longitudinal bars, (A_c_)is the total concrete area (A_c_ = b*h), (A_st_) is the area of ​​one stirrup leg, (p_t_) is the perimeter of the stirrup, and (s_t_)is the distance between the stirrup centers. The end areas of the beam (250 mm long for each end) were further reinforced. Internally, 10mm diameter closed stirrups by Yusuf, et al.^20^.

One beam was used as a control beam (Bcon), while four other beams (BV1 – BV4) were reinforced with Ø10 mm U-shaped steel bars embedded in surface channels filled with SikaDur-31 CF high-strength epoxy adhesive.

The effect of three main variables on the torsional behavior of braced beams was studied as shown in Table 1.

- The overlap length between the two outer beam branches (Lo).
- The spacing between the beams (S).
- The presence of an internal hook at the beam ends.

**Table 1:** Program details for tested beams^20^.

No	Name	Dimensions (mm)			Reinforcement		Strengthening			Notes
		W	H	L	Longitudinal	Stirrups	Type	Lo (mm)	S (mm)	
1	B con	150	300	1500	2 Ø 12 T&B	4 ɸ 10 closed stirrups + 5 ɸ 8 opened stirrups	Without strengthening			
2	BV1						Steel bars Ø 10	100	200	–
3	BV2							100	125	
4	BV3							150	200	
5	BV4							100	125	hook

The details of the brace systems were as follows:

- Beam BV1: Lo = 100 mm, S = 200 mm.
- Beam BV2: Lo = 100 mm, S = 125 mm.
- Beam BV3: Lo = 150 mm, S = 200 mm.
- Beam BV4: Lo = 100 mm, S = 125 mm, with a 50 mm internal hook.

### Experimental program results

The loading was performed using a special loading device designed to produce pure torsional moments without generating bending moments. This was accomplished by means of a 500 mm long steel loading arm attached to the ends of the beam. Low-voltage displacement devices (LVDTs) were used to accurately record the torsion angle during the loading phases.

Experimental results showed a clear improvement in the performance of the reinforced beams compared to the control beam. The maximum torsional moment increased by 36% to 112% depending on the reinforcement system, while the maximum torsion angle increased by 83% to 267%. The addition of the hook in the BV4 system significantly improved performance due to the increased bonding of the reinforcement with the concrete section, providing additional resistance similar to the effect of internal cantilevers.

In terms of crack patterns, it was observed that the reinforced beams exhibited regular, fine hairline cracks at a 45° angle, while the control beam was characterized by wide, rapidly propagating radial cracks Yusuf, et al.^20^.

Accordingly, this experimental study Yusuf, et al.^20^, serves as a basic reference for constructing the proposed numerical model, in terms of specimen dimensions, material properties, loading conditions, and the nature of failure. This allows for accurate numerical simulations to validate the model and compare its results with experimental results at various loading phases.

The Abaqus program will be used to model the beams implemented in the numerical program, and the numerical results will be compared with the experimental results to obtain a model that matches the actual model. This will be used to study several parametric studies, such as:

- Changing the overlap of external reinforcement stirrups.
- Hybridization of different reinforcement systems, such as NSM and EBR, by adding steel.

mesh with different layers.

Therefore, we must develop a finite element model to model the materials and determine their properties to obtain a sound model.

## Numerical investigation

### Finite element model

#### Geometric modeling technique

Recent advances in computer science have expanded the scope of numerical simulation, making finite element analysis (FEA) an effective and cost-effective method compared to expensive and time-consuming experimental testing, despite some limitations resulting from model simplifications. In this research, Abaqus (Simulia, 2020) was used to perform numerical simulations, clarifying the basic parameters used in the modeling.

A numerical model was constructed using three-dimensional elements representing all components of the beam.

**Fig. 2:**
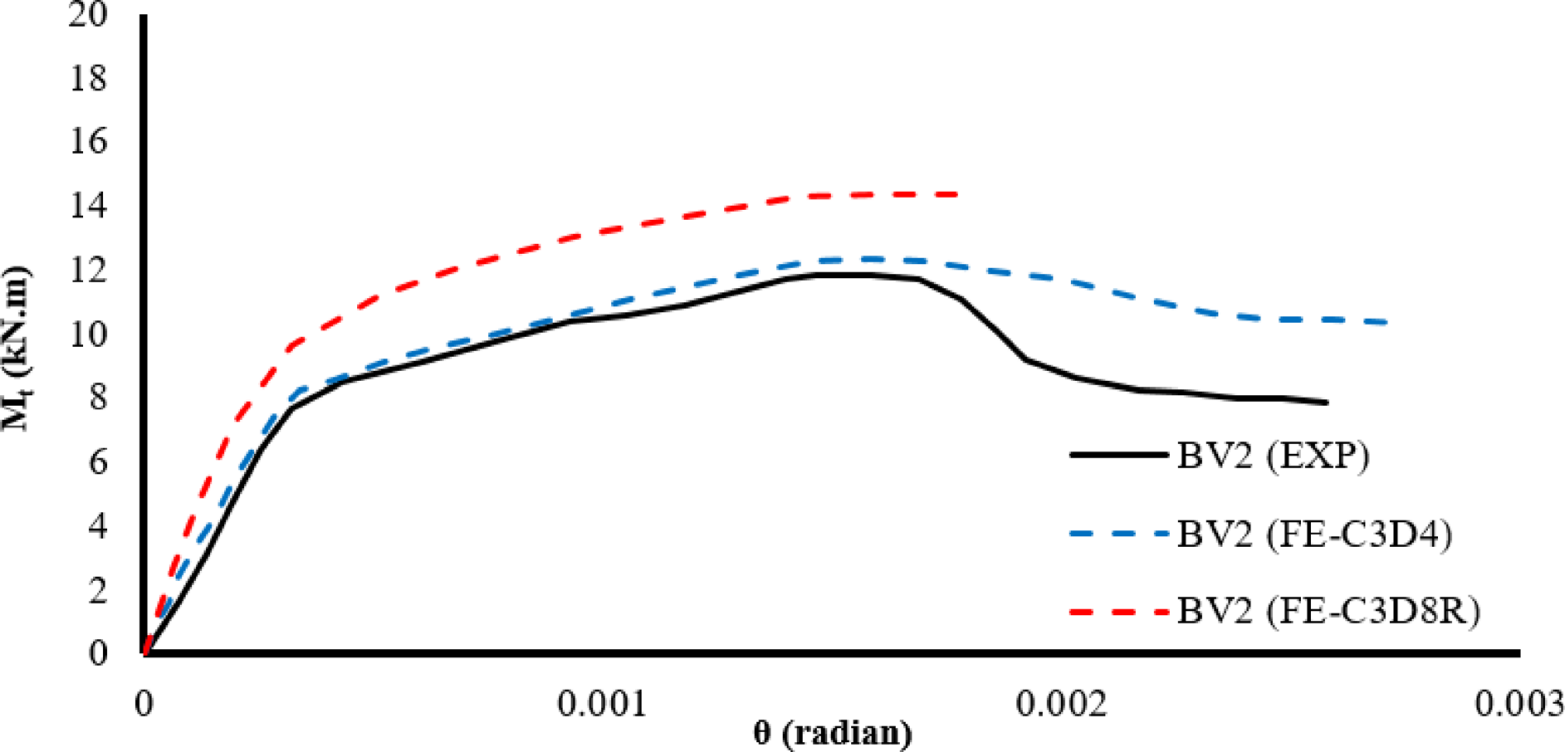
The effect of element type on the behavior of beam BV2.

**Fig. 3:**
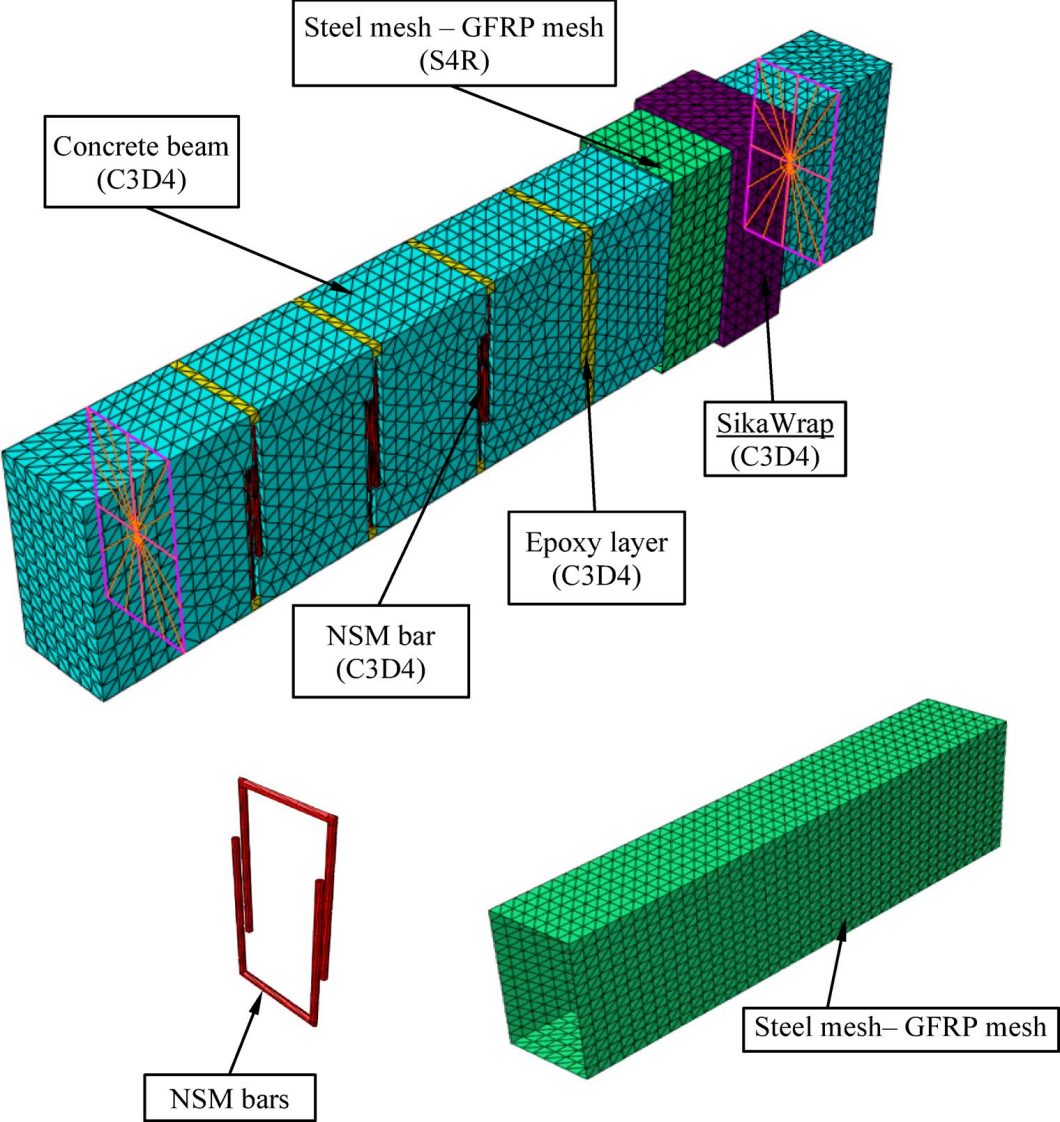
Finite Element Model.

After comparing elements C3D4 and C3D8R, it was found that the optimal model, compared to the experimental results, was element C3D4, as shown in Fig. 2, with steel bars fixed near the surface (NSM), an epoxy layer inside the channels, and a SikaWrap coating. Element S4R was used to represent the mesh layers, whether made of glass fiber-reinforced plastic (GFRP) or steel as shown in Fig. 3.

**Fig. 4:**
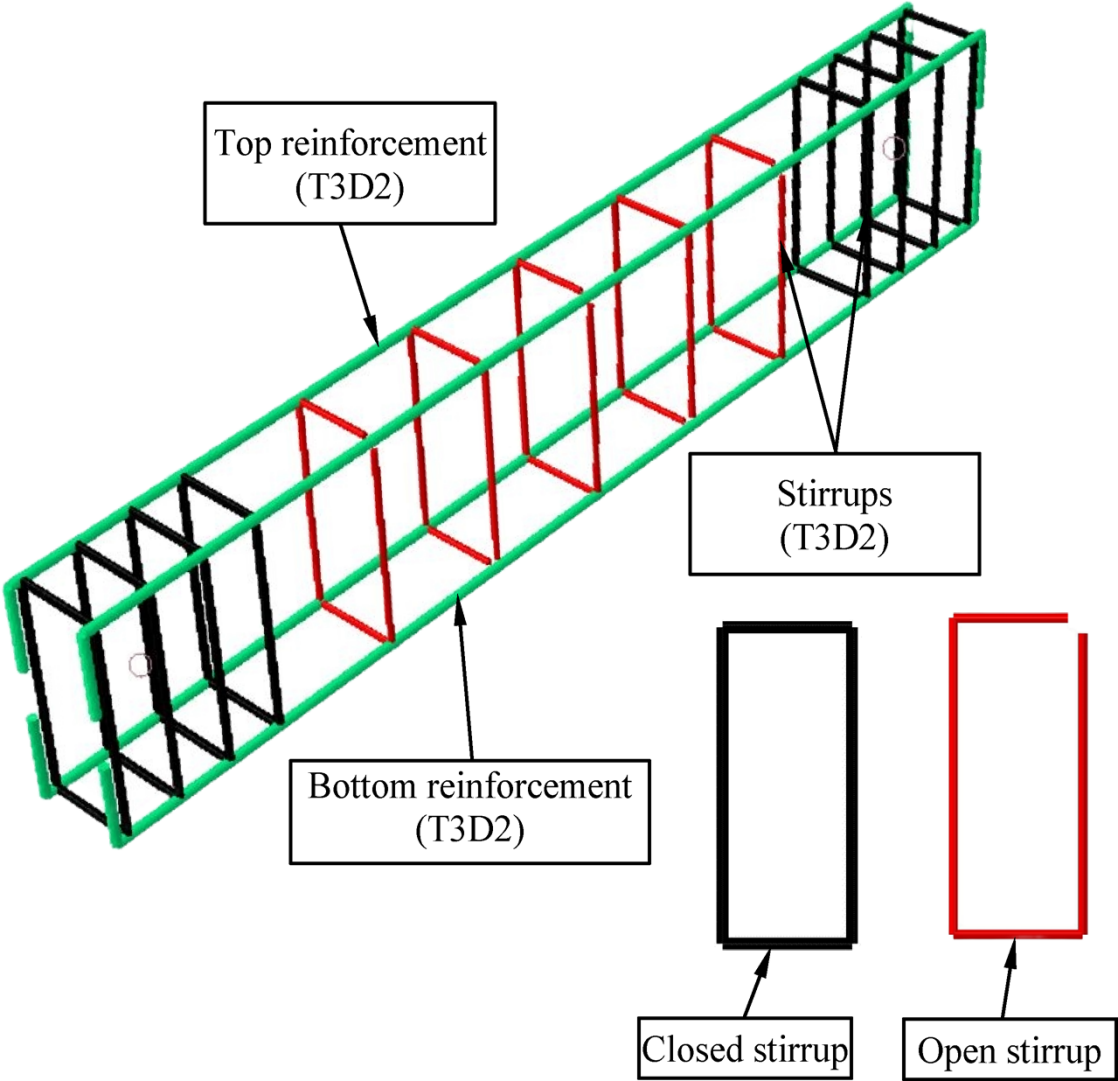
Finite Element Model for Reinforcement.

While the element T3D2 was used to represent the internal longitudinal reinforcement and beams, as implemented in many researches. The open beams were designed with an unclosed part longer than the web size, while the closed beams were implemented as complete circular rings^22–25^ , as shown in Fig. 4.

### Numerical models of test beams

**Fig. 5:**
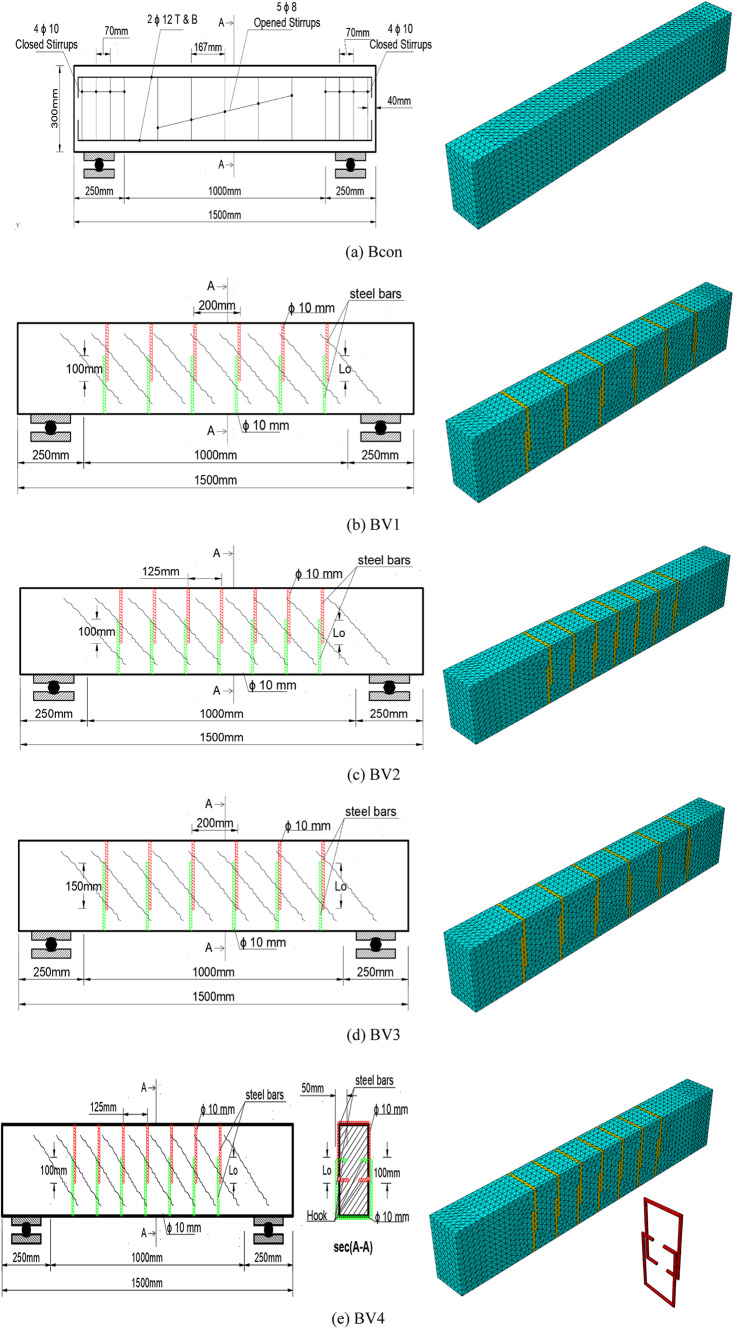
The numerical models of tested beams (**a**) Bcon, (**b**) BV1, (**c**) BV2, (**d**) BV3, (**e**) BV4.

Below is a presentation of the finite element model developed for the beams, showing the complete set of numerical models corresponding to all the tested beams. These models formed the basis for the verification study and subsequent parametric analyses. as shown in Fig. 5.

### Interactions between components

The behavior of interfaces between different materials plays a crucial role in the accuracy of the structural response of numerical models. In this research, Perfect bonding was assumed for the interfaces that showed no signs of separation during laboratory tests, including the concrete interface with internal reinforcement, the concrete interface with SikaWrap, and the concrete interface with epoxy within the grooves. In contrast, the NSM bar interface with epoxy was modeled using a cohesive zone model to simulate the slip and gradual separation behavior, as it is the most affected and sensitive interface under torsional loads^24^.

**Fig. 6:**
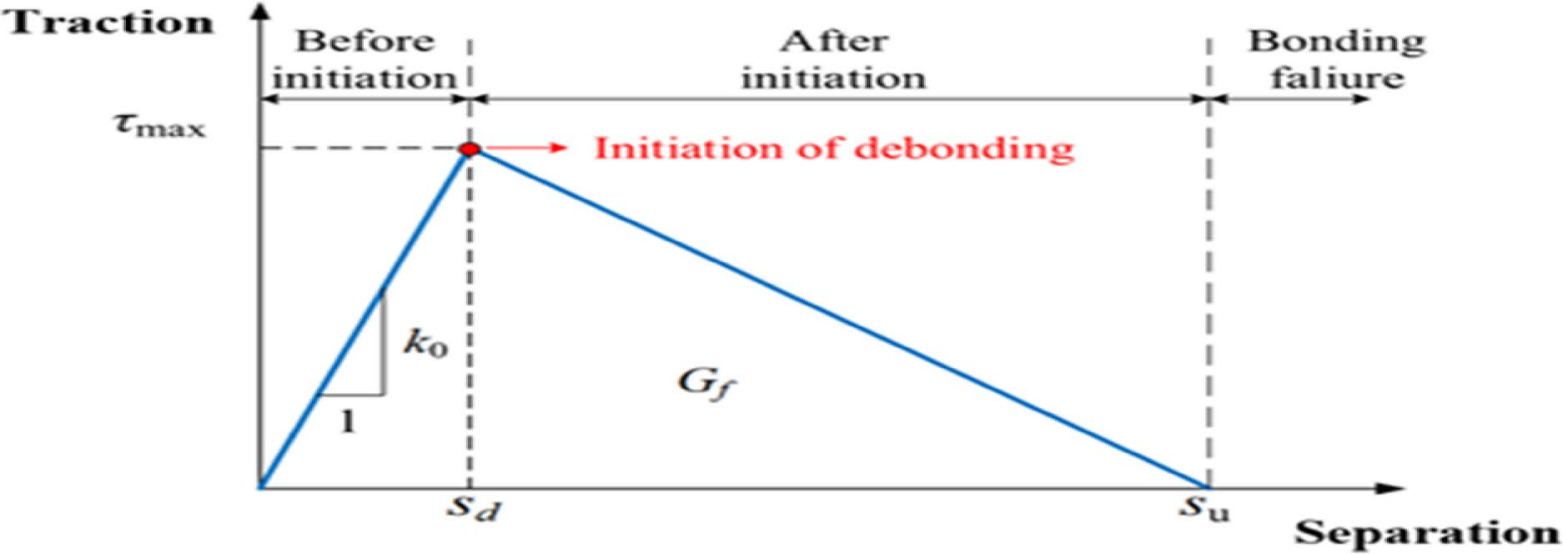
Constitutive relationships of the bilinear traction–separation model^24^.

. For the epoxy-filled grooves, the model assumed perfect bonding with the surrounding concrete, consistent with experimental results that showed no detachment between the two materials. In contrast, the NSM bars-epoxy interface represented a more complex case, where a cohesive damage model was used to simulate the detachment process observed in some specimens. This law consists of three basic stages: a linear elastic response up to the maximum bond strength, followed by damage initiation when that strength is exceeded, and then gradual debonding controlled by the fracture energy that determines the softening curve and the gradual loss of load-transfer capacity. This model enabled the accurate reproduction of early debonding failure patterns, as shown schematically as shown in Fig. 6.

### Boundary conditions and loading method

**Fig. 7:**
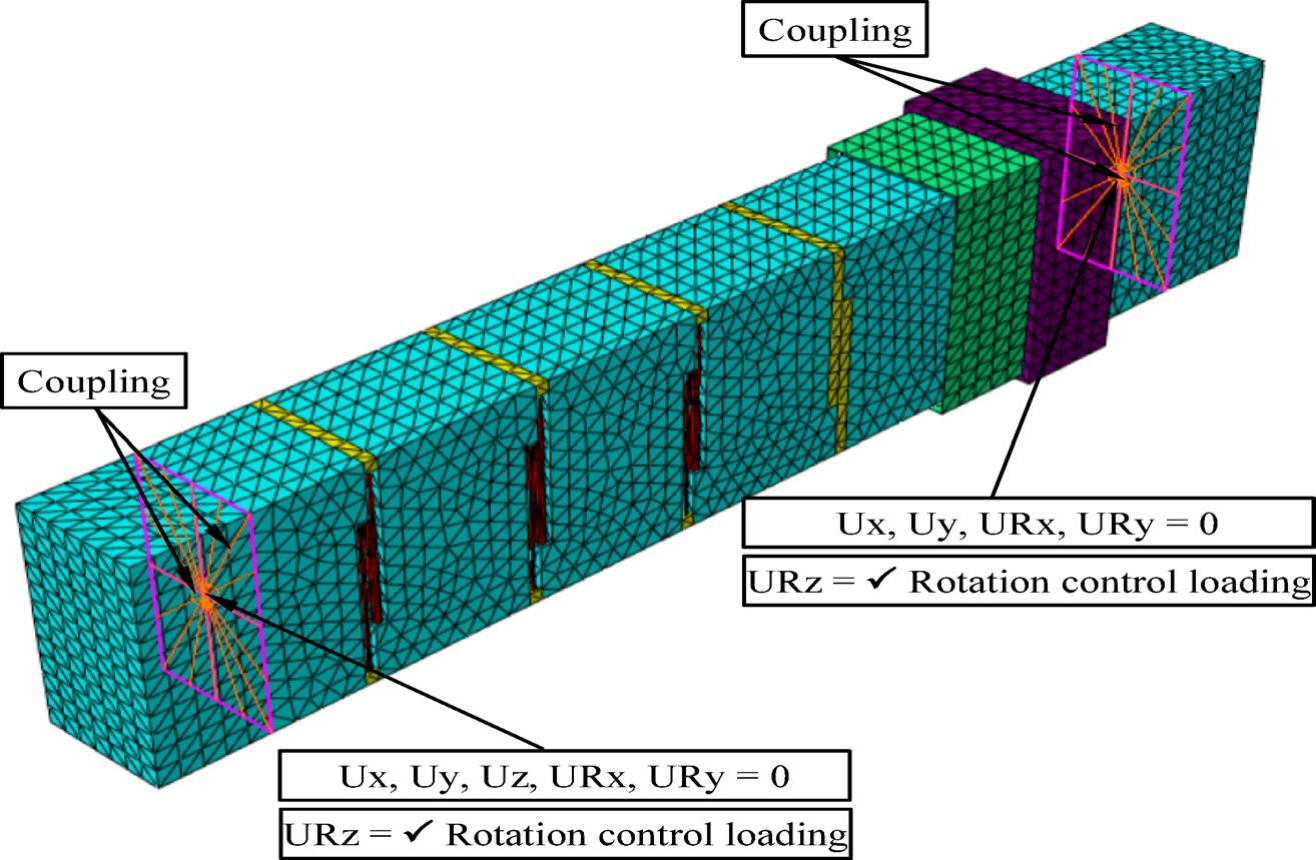
Boundary conditions and loading method.

Torsional loading was applied through rotation control about the longitudinal axis, while all other translational and rotational degrees of freedom were appropriately constrained using coupling conditions to ensure a uniform distribution of torsional moment at the beam ends. The adopted boundary conditions were carefully defined to replicate the experimental setup and to guarantee the development of a pure torsional state without inducing any unintended bending moments or shear forces. At the mid-span, all displacements and rotations were released, except for the rotational degree of freedom about the longitudinal axis (URz), whereas at the opposite end, longitudinal displacement (Uz) was allowed while the remaining degrees of freedom were restrained. Opposing torques were then imposed at both ends through controlled rotations, ensuring uniform torsional stress along the beam length. This configuration effectively eliminated parasitic effects and provided an accurate numerical representation of the pure torsion conditions observed experimentally, as shown in Fig. 7.

### Modeling of materials

Material properties in the numerical model were determined to reflect realistic behavior under loads. An elastic–plastic model with stiffness was used to represent the behavior of the reinforcing steel, steel bars, and steel mesh. This model expresses the elastic response of the material until yielding, followed by a transition to a plastic phase with increasing stress.

The GFRP mesh was modeled as elastic until fracture to simulate its brittle behavior.

The epoxy coating and SikaWrap were modeled using a traction-segregation damage law model, which relies on the relationship between tensile and segregation forces to simulate damage initiation and progression at interfaces. This model helped represent the gradual segregation behavior between components in a realistic manner consistent with experimental observations, particularly in weak areas at the reinforced concrete interface.

#### Concrete

Concrete was modeled using the Concrete Damaged Plasticity (CDP) model for its proven ability to monitor stiffness degradation, tensile cracking, and subsequent softening, which are dominant mechanisms in torsional behavior. Because torsional failure in reinforced concrete beams is controlled primarily by diagonal tensile stresses rather than pure compression, the CDP framework provides a more realistic representation than elastic or diffuse cracking models.^24^, which relies on continuous damage mechanics to represent the nonlinear behavior of concrete in compression and tension. The model takes into account stiffness deterioration due to cracking and buckling, defining key parameters such as the expansion angle (37°), deflection (0.1), shape factor (0.667), and the ratio of biaxial to uniaxial compressive strength (1.16). A small viscosity coefficient (0.00001) is also used to ensure the stability of numerical solutions. This model allows for the simulation of crack development and gradual collapse in tension and compression zones, reflecting the real behavior of concrete under torsion,

The adopted values ​​for these parameters are summarized in Table 2, consistent with those reported in scientific literature. Equations (1)–(7) define the constitutive relationships governing the stress–strain response in tension and compression, as well as the evolution of damage variables. These formulations allowed the finite element (FE) model to monitor both crack initiation and the gradual deterioration of stiffness in the concrete matrix^26^.

**Table 2:** CDP model variables used to model nonlinear behavior of concrete^24^.

Dilation angle (ψ)	Eccentricity (e)	Shape parameter (K_c_)	Maximum compression axial/biaxial (*f*_*bo*_*/f*_*co*_)	Viscosity (μ)
37°	0.1	0.667	1.16	0.00001

The compressive stress–strain relationship of concrete was defined using a modified Hognestad model as shown in Eqs. (1–3), calibrated based on the experimentally measured compressive strength. Tensile behavior was modeled as linear elastic up to the cracking stress, followed by a tension softening branch governed by fracture energy to ensure mesh objectivity. The fracture energy values were selected in accordance with published numerical studies on torsional behavior of RC members and were calibrated to reproduce the experimentally observed cracking torque and post-cracking stiffness degradation^24,26^.

1 $$\frac{{f}_{c}}{{f}_{c}^{,}}=\frac{n \left[\frac{{\varepsilon }_{c}}{{\varepsilon }_{0}}\right]}{n - 1+{\left[\frac{{\varepsilon }_{c}}{{\varepsilon }_{0}}\right]}^{nk}}$$

2 $$n=0.8+ \frac{{f}_{c}^{,}}{17.25}$$

3 $$k=0.67+ \frac{{f}_{c}^{,}}{62} \ge 1$$

4 $$\varepsilon_{c}^{ - in} = \varepsilon_{c} - \varepsilon_{0c}^{ - el} = \varepsilon_{c} - \frac{{f_{c} }}{{E_{0} }}$$

5 $$\varepsilon_{c}^{ - pl} = \varepsilon_{c}^{ - in} = \frac{{d_{c} }}{{\left( {1 - d_{c} } \right)}}\frac{{f_{c} }}{{E_{0} }}$$

6 $$\varepsilon_{c}^{ - ck} = \varepsilon_{t} - \varepsilon_{0t}^{ - el} = \varepsilon_{t} - \frac{{\sigma_{t} }}{{E_{0} }}$$

7 $$\varepsilon_{t}^{ - pl} = \varepsilon_{t}^{ - ck} = \frac{{d_{t} }}{{\left( {1 - d_{t} } \right)}}\frac{{\sigma_{t} }}{{E_{0} }}$$

where ${f}_{c}$​: the compressive stress corresponding to strain ${\varepsilon }_{c}$​, ${f}_{c}^{,}:$ the cylinder compressive strength, ${\varepsilon }_{0}$​: the strain corresponding to the peak stress ${f}_{c}^{,}.$ ${\varepsilon }_{c} , {\varepsilon }_{t}$: total compressive and tensile strains, respectively. ${\sigma }_{t}$​: tensile stress. ​${E}_{0}$: initial (undamaged) elastic modulus of the material. $\varepsilon _{{0c}}^{{ - el}}$, $\varepsilon _{{0t}}^{{ - el}}$: elastic components of the compressive and tensile strains, respectively. $\varepsilon _{c}^{{ - in}}$, $\varepsilon _{t}^{{ - ck}}$: inelastic (or cracking) components of the compressive and tensile strains, respectively. $\varepsilon _{c}^{{ - pl}}$, $\varepsilon _{t}^{{ - pl}}$: plastic components of the compressive and tensile strains, respectively. ​${d}_{c}$, ${d}_{t}$: scalar damage variables in compression and tension, respectively, with values ranging between 0 (undamaged) and 1 (fully damaged)^26^.

#### Reinforcement steel and NSM bars

The longitudinal reinforcement, stirrups, NSM bars, and steel mesh were represented in the numerical model using an elastic–plastic model with isotropic hardening. This model assumes that the material behaves linearly elastically until it reaches a yield stress. Beyond this stress, plastic deformation begins, accompanied by strain hardening, enabling the material to withstand additional loads with increased plastic strain. This representation is very suitable for steel used in reinforcement, as it accurately reflects the realistic behavior of strength gain after yielding as observed in experimental tests.

**Fig. 8:**
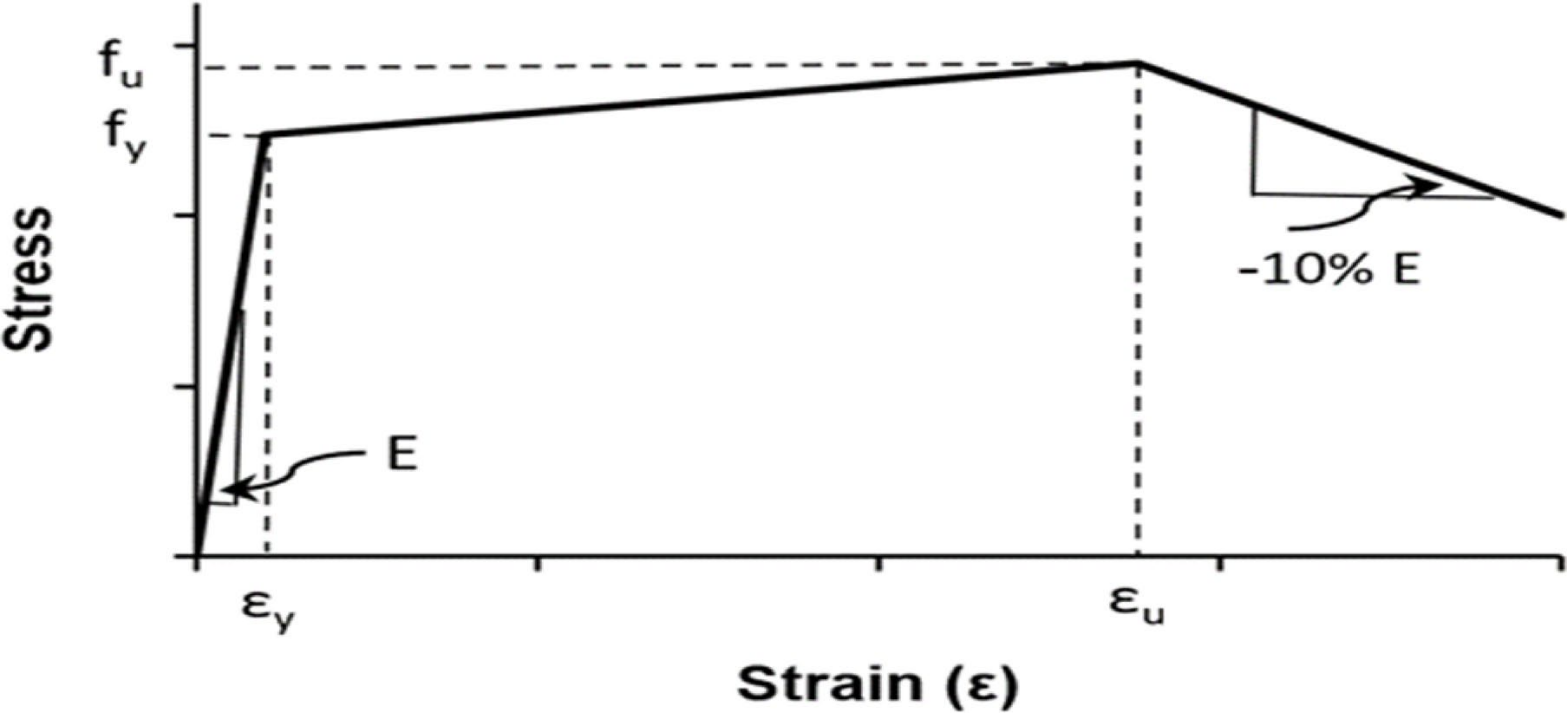
Elastic–plastic material model with hardening^23^.

Figure 8, illustrates the stress–strain relationship according to this law, which can be expressed mathematically by Eq. (8). The hardening branch was of particular importance in determining the ductility of reinforcement and its ability to redistribute stresses under the influence of torsional loads^23^.

8 $${f}_{s}=\left\{\begin{array}{c}{E}_{s} {\varepsilon }_{s} {\varepsilon }_{s}\le {\varepsilon }_{y}\\ {f}_{y}+ \frac{{\varepsilon }_{s}-{\varepsilon }_{y}}{{\varepsilon }_{u}-{\varepsilon }_{y}} \left({f}_{u}-{f}_{y}\right) {\varepsilon }_{y}<{\varepsilon }_{s}\le {\varepsilon }_{u}\\ {f}_{u}-0.1{E}_{s}\left({\varepsilon }_{s}-{\varepsilon }_{u}\right) {\varepsilon }_{s}>{\varepsilon }_{u}\end{array}\right.$$

#### Epoxy and Sikawrap layers

The epoxy and Sika rep layer were modeled using the "damage to traction and shearing laws" approach. This model is commonly used to simulate the mechanical response of bonded interfaces, especially in bonded materials. This model specifies the traction and shearing relationship that characterizes the interface behavior under loading, including damage initiation and progression, as shown in Fig. 6. The properties of the epoxy materials were determined from their datasheets and their values ​​were entered into the model, as described in the practical article^20^.

### Parametric study and sensitivity analysis

The criteria studied were not assumed randomly, but were chosen based on empirical observations and then calibrated through sensitivity analyses. This approach ensures that the adopted numerical criteria reflect actual structural behavior rather than ideal assumptions, and that they are based on the same principle of the practical component to determine when the model is reliable and the effect is significant.

#### Dilation angle calibration (ψ)

**Fig. 9:**
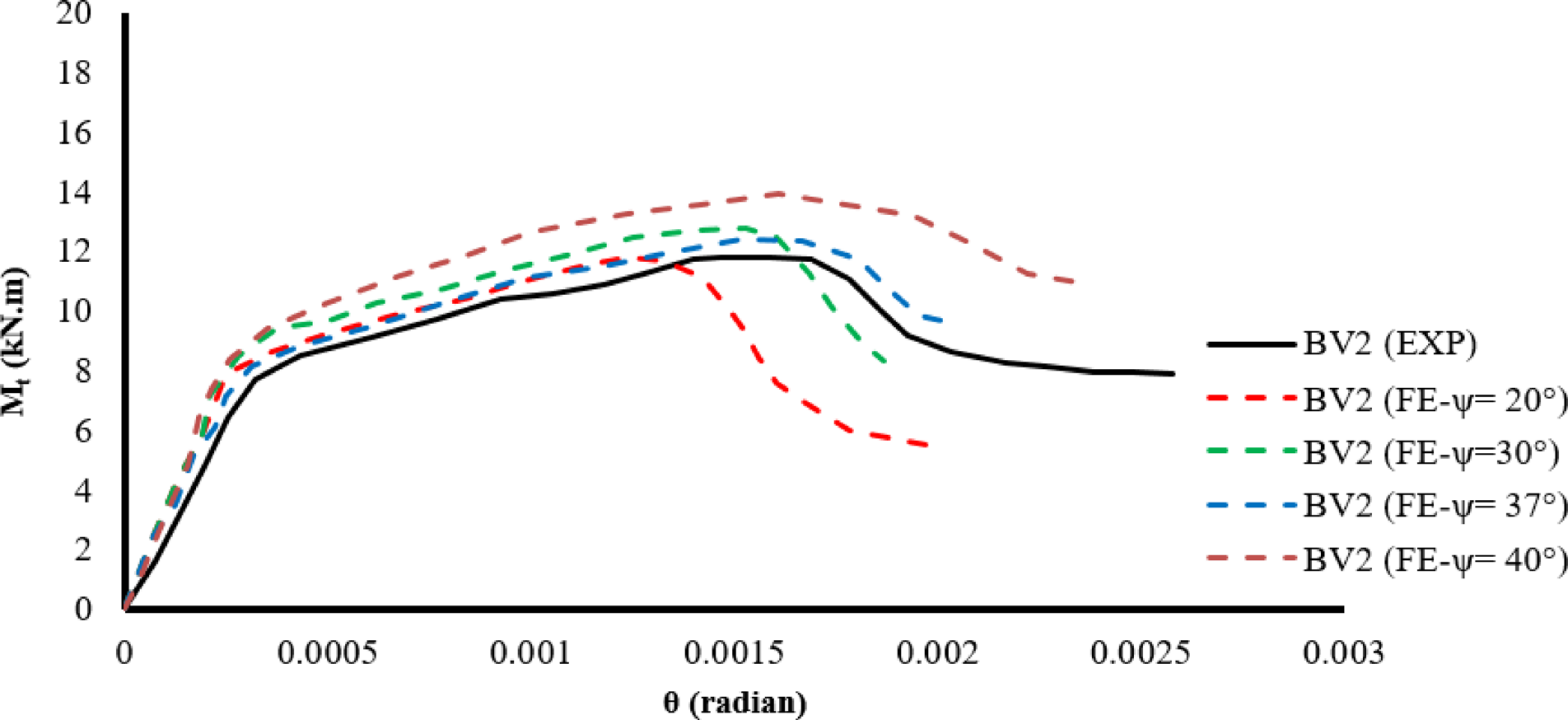
The effect of the dilation angle value on the behavior of beam BV2.

A parametric study was conducted on beam BV2 to evaluate the effect of the extension angle on the torsional response. Various extension angle values ​​were studied, ranging from 20 to 40 degrees, as shown in Fig. 9. The results showed that an extension angle of 37 degrees provides a suitable balance for monitoring the beam’s behavior. Angles lower than this value result in a brittle response, as observed from the torque-to-torsion angle relationship, while angles higher than 37 degrees exhibit an elastic behavior, which deviates from the experimentally observed response. In addition, a sensitivity analysis was conducted on the mesh size used in the finite element modeling.

The dilation angle ψ = 37° was therefore adopted as a calibrated value rather than a predefined constant, as it provided the closest agreement with experimental torque–twist responses while avoiding unrealistically brittle or overly elastic numerical behavior.

#### Mesh sensitivity analysis

**Fig. 10:**
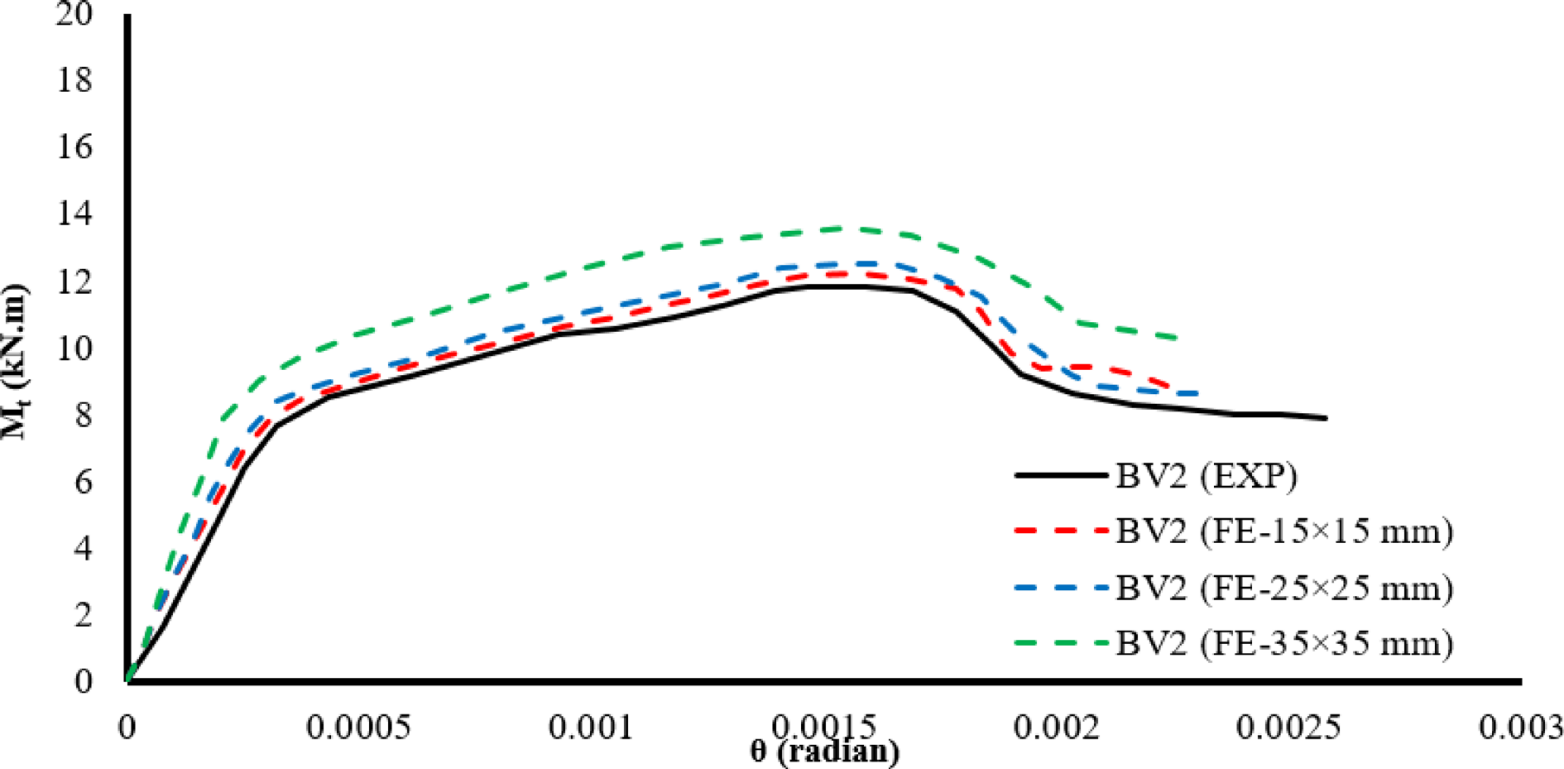
The effect of mesh size on the behavior of beam BV2.

Three mesh sizes were studied: 15 × 15 mm, 25 × 25 mm, and 35 × 35 mm, as shown in Fig. 10. The 25 × 25 mm mesh was found to provide a computationally efficient alternative without compromising accuracy, providing results comparable to those obtained using the finer 15 × 15 mm mesh, while reducing computation time by up to 30%. On the other hand, the 35 × 35 mm models exhibited an excessively stiff response, which does not accurately reflect the actual behavior of the tested beams.

Table 3 discusses mesh sensitivity analysis: the effect of element size on torsion resistance accuracy, computational cost, and numerical convergence behavior.

**Table 3:** Mesh sensitivity quantitative comparison.

Mesh size (mm)	Tu error (%)	CPU time (relative)	Convergence
15 × 15	2.1	1.00	Stable
25 × 25	3.8	0.70	Stable
35 × 35	9.5	0.45	Over-stiff

### Numerical validation

Validating the finite element model was a key step in assessing its accuracy in simulating the actual behavior of concrete beams under torsion. In Abaqus, the Damage in Tensile (DAMAGET) parameter was used to track crack development within the concrete. This parameter represents the damage range from 0 (no damage) to 1 (complete tensile failure). Since torsional loading in concrete beams results in diagonal tensile stresses that cause diagonal cracks, using DAMAGET was an effective way to track crack initiation and propagation during numerical simulations. Furthermore, the 25 × 25 medium mesh was found to yield very similar results to the experimental results in terms of maximum torque and maximum torsion angle, as well as in a shorter time compared to other meshes.

### Comparison of the experimental and finite element findings

#### cracking patterns

**Fig. 11:**
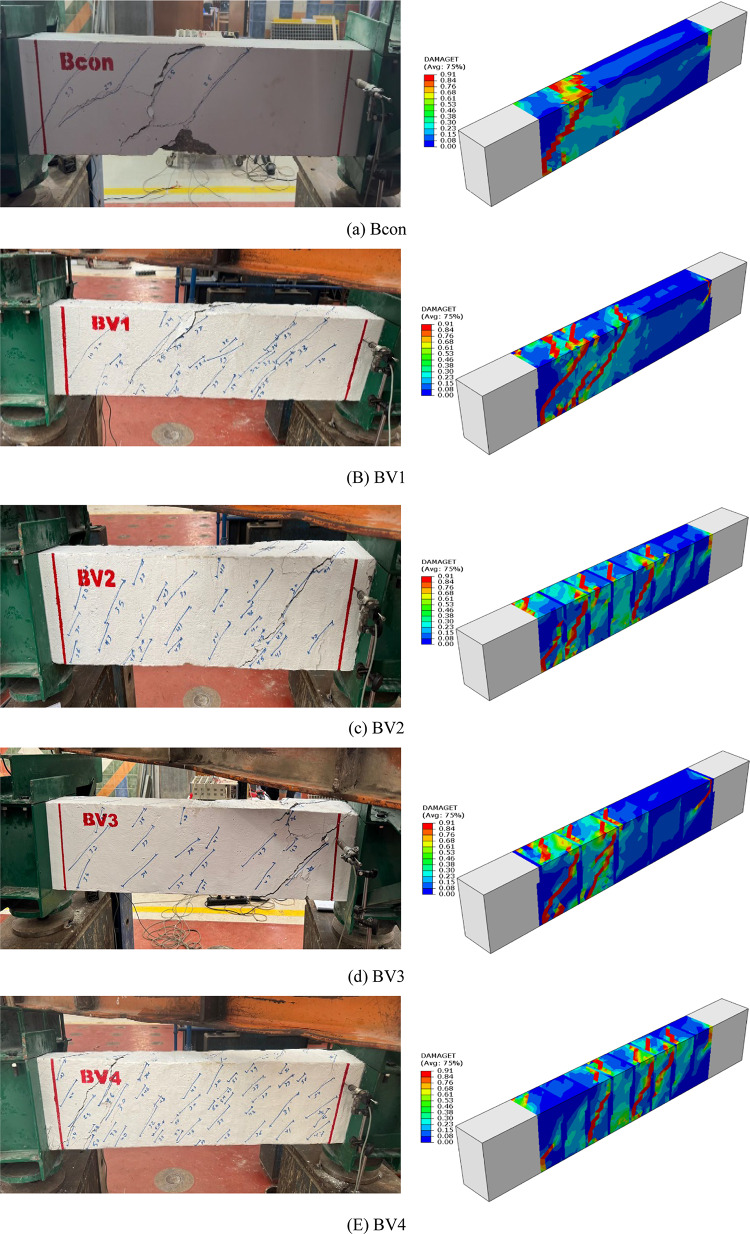
Comparison of experimental and numerical collapse patterns (**a**) Bcon, (**b**) BV1, (**c**) BV2, (**d**) BV3, (**e**) BV4.

A comparison of the experimental results with those derived from the numerical model, as shown in Fig. 11, showed remarkable agreement in the locations and orientations of diagonal cracks, particularly in the shear zones subject to the highest torsional stresses. The numerical model successfully reproduced crack development with high accuracy, from initial cracking to advanced crack patterns, reflecting a realistic representation of the failure mechanism. The crack propagation and distribution between the actual specimens and the numerical model also showed strong consistency, confirming the accuracy of the model setup and the reliability of the simulation approach.

This consistency between the overall behavior of the beams (in terms of torque response and torsion angle) and the local failure mechanisms (in terms of crack initiation and propagation) enhances the reliability of the developed model. This indicates that the constitutive models, interaction laws, and calibration strategies adopted were appropriate and accurate, enabling the model to be used with confidence for conducting extended parametric studies beyond laboratory testing.

Numerical validation was evaluated by comparing the torque-versus-torsion curves between the experimental results and those derived from numerical analysis, as shown in Table 4. The results showed excellent agreement between the two sets, with the difference in ultimate torsional moment not exceeding 5% and the corresponding rotation angle of 3% at the maximum stage, reflecting the accuracy of the stiffness and toughness properties under torsional loading. The numerical initial stiffness matched the experimental response well, indicating an accurate representation of the elastic properties and boundary conditions. The model also demonstrated a high ability to predict cracking moments and corresponding torsional values, confirming the effectiveness of the ductile damaged concrete (CDP) model in representing tensile crack initiation. Furthermore, the numerical values ​​of maximum torque (M_t_) showed close agreement with the experimentally measured values, while the differences remained within acceptable limits according to previous studies.

**Table 4:** Numerical versus experimental results.

Stage	Ultimate					
beams ID	M_t_ (kN.m)			θ_t_ (radian)		
	EXP	FE	FE/ EXP	EXP	FE	FE/ EXP
Bcon	7.3	7.2	0.99	0.0006	0.0007	1.17
BV1	9.9	10.1	1.02	0.0012	0.0011	0.92
BV2	11.8	12.6	1.07	0.0015	0.0015	1
BV3	10.8	12.2	1.13	0.0019	0.0019	1
BV4	15.2	15.9	1.05	0.0021	0.0022	1.05
Avg			1.05			1.03
SD			0.053			0.092
CoV			5.00%			9.00%

EXP: Experimental; FE: Finite element; Avg: Average; SD: Standard deviation; CoV: Coefficient of variation

**Fig. 12:**
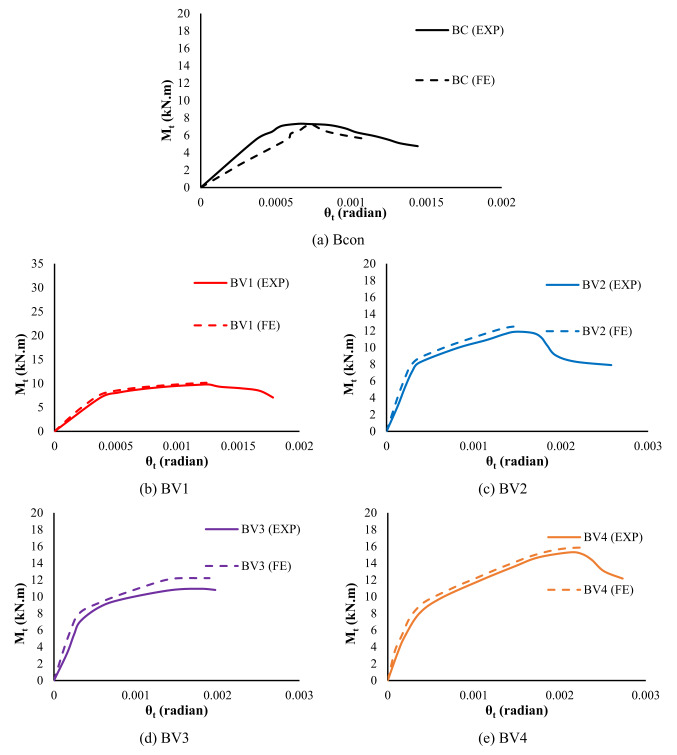
Comparison of experimental and numerical torque–twist responses for the tested beam (**a**) Bcon, (**b**) BV1, (**c**) BV2, (**d**) BV3, (**e**) BV4.

Although some minor variations in post-peak behavior were observed due to physical factors that are difficult to simulate numerically (such as local defects in real samples), the numerical model maintained a high accuracy in representing the torque-torsion curves, as shown in Fig. 12, confirming its reliability. Thus, it can be said that this strong agreement between the experimental and numerical results not only enhances confidence in the adopted modeling strategy but also forms a solid basis for expanding the scope of the study and conducting advanced analyses of the influence of basic design parameters beyond the limits of practical tests.

## Parametric study

### Vertical strengthening

#### Change in overlap length in NSM external stirrups

**Fig. 13:**
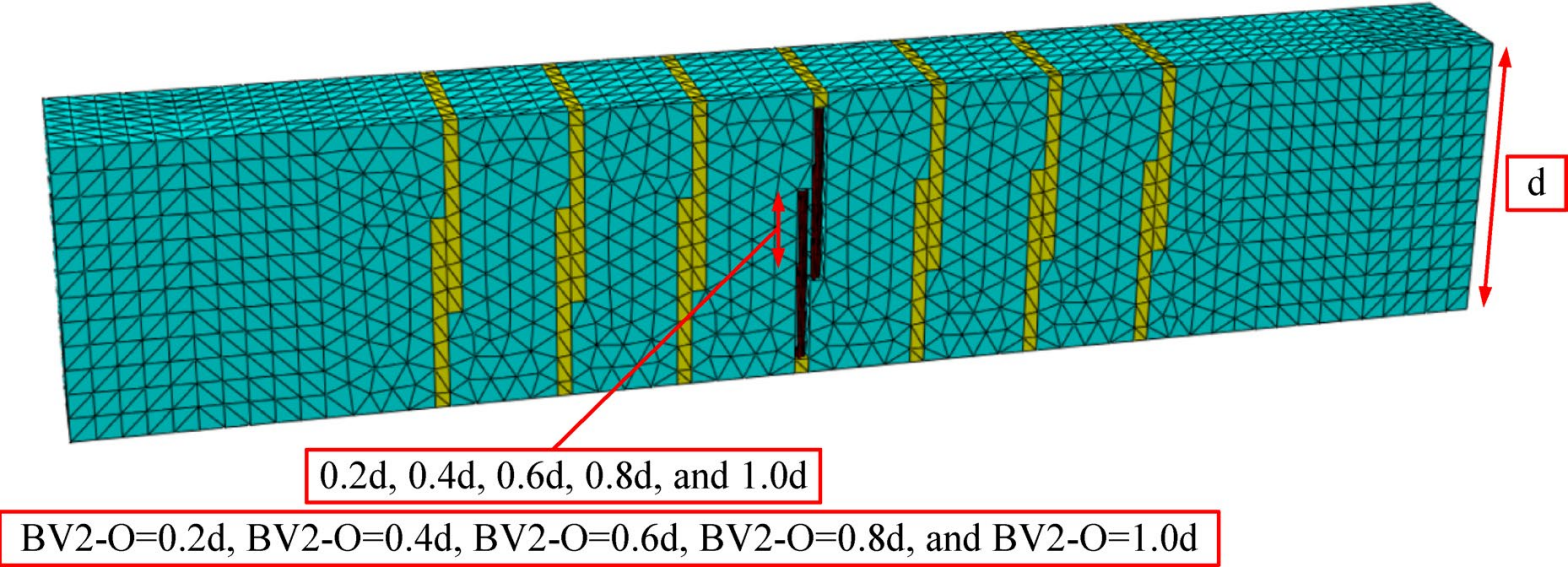
Beams of the parametric study with variable overlap length.

A benchmark study was conducted to evaluate the effectiveness of near-surface external stirrups (NSM) in strengthening reinforced concrete beams with open internal stirrups under torsional loading. The reference beam (BC), which contained open stirrups, exhibited a maximum moment capacity of 7.2 kNm, reflecting its inherent weakness in torsional resistance. To improve its performance, beam BV2 was chosen as the strengthening configuration, in which NSM external stirrups were used. Different overlap lengths (O), expressed as a percentage of the total beam depth (d), were studied to evaluate their effect on torsional resistance. The tested overlap ratios were 0.2d, 0.4d, 0.6d, 0.8d, and 1.0d, corresponding to beams BV2-O = 0.2d, BV2-O = 0.4d, BV2-O = 0.6d, BV2-O = 0.8d, and BV2-O = 1.0d, respectively as shown in Fig. 13.

##### Effect of overlap length of external NSM stirrups

**Fig. 14:**
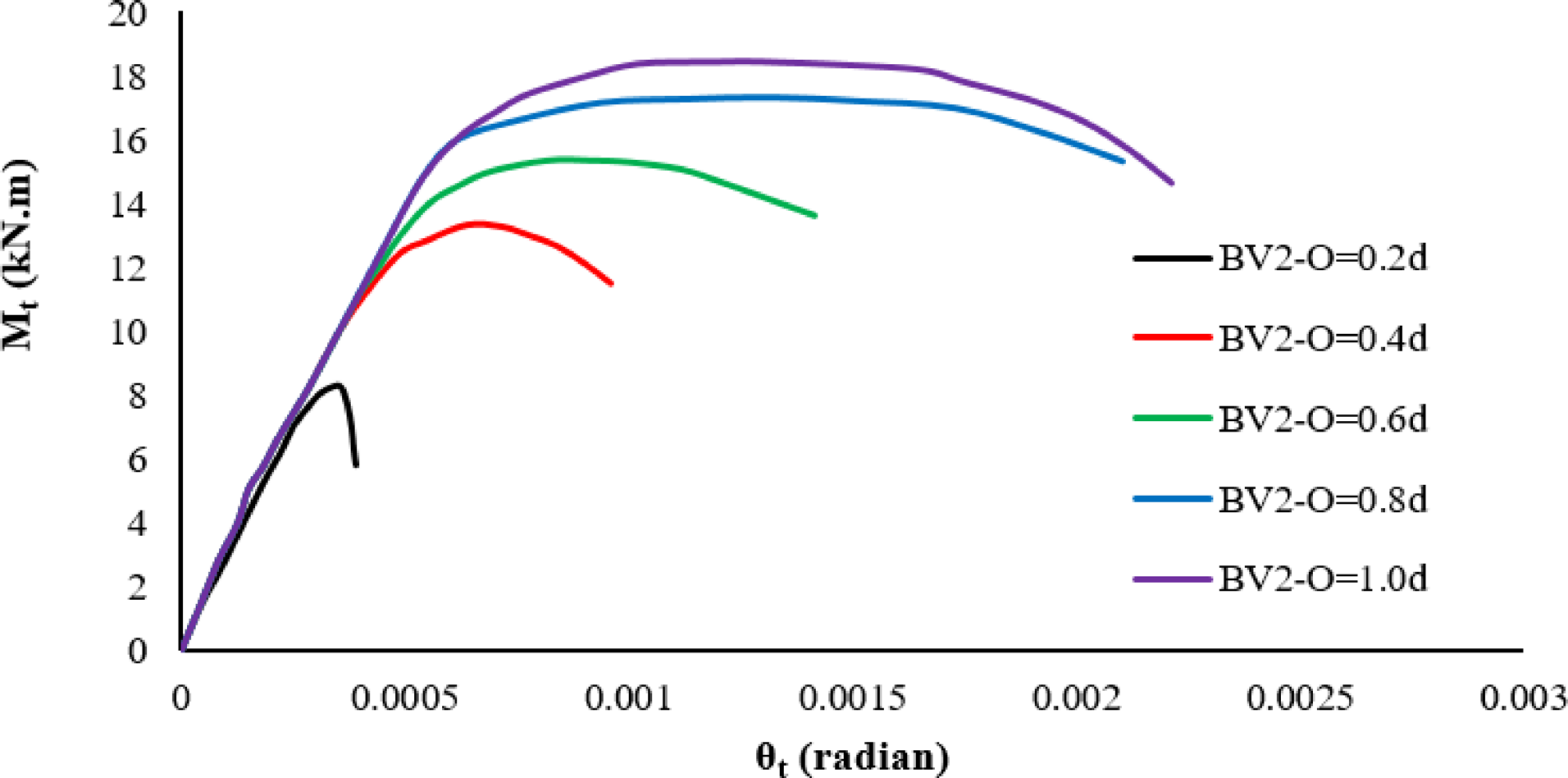
Twist angle–torsional moment curves of the tested beams.

**Fig. 15:**
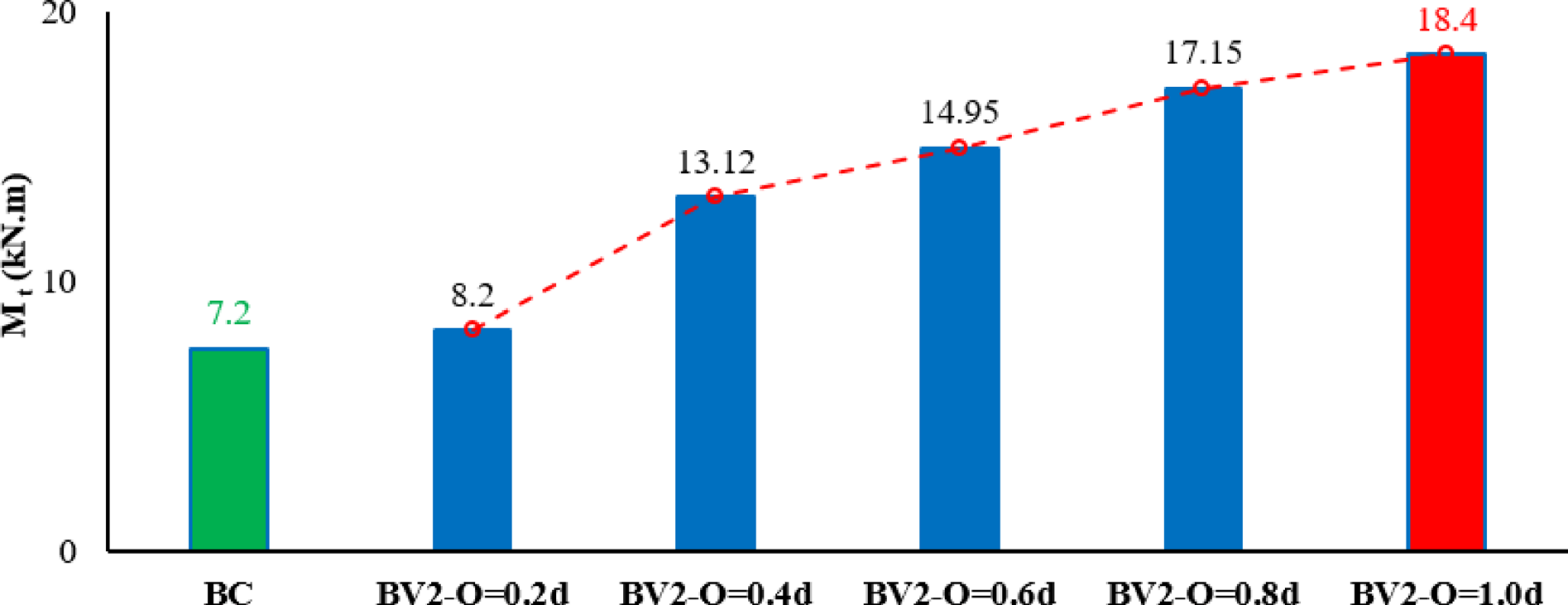
Effect of overlap length on maximum torsional moment.

After applying the variables to the designed beam model, the results, as shown in Table 5, showed that even a short overlap length of 0.2d resulted in a modest increase in the torsional strength (approximately 14% higher than BC). However, although the corresponding torsional angle decreased by 57%, reflecting that the beam’s shearing compared to the used overlap length resulted in faster failure and reduced ductility, when the overlap was extended to 0.4d, the torsional capacity increased significantly by more than 82% compared to the reference beam, while the torsional angle remained unchanged. Further increases in overlap length continued to enhance the torsional strength. Increasing the overlap to 0.6 d increased the torque capacity by 110% with a slight increase in the torsional angle of 14%. Increasing the overlap length to 0.8 d increased the torsional torque by 138% while the torsional angle increased by 86%. Finally, applying the maximum overlap length of 1.0 d increased the maximum torsional torque by 156% and the torsional angle by 43%. The results are represented by a curve as shown in Figs. 14, 15.

**Table 5:** Results of changes in the overlap length.

Beams ID	Overlap length	M_t_ (kN.m)	Inc%	θ_t_ (radian)	Inc%
Bcon	0	7.2	0	0.0007	0
BV2	0.2d	8.2	1.14	0.0003	-0.57
	0.4d	13.12	1.82	0.0007	0
	0.6d	14.95	2.1	0.0008	1.14
	0.8d	17.15	2.38	0.0013	1.86
	1.0 d	18.4	2.56	0.001	1.43

##### Numerical failure modes of beams

**Fig. 16:**
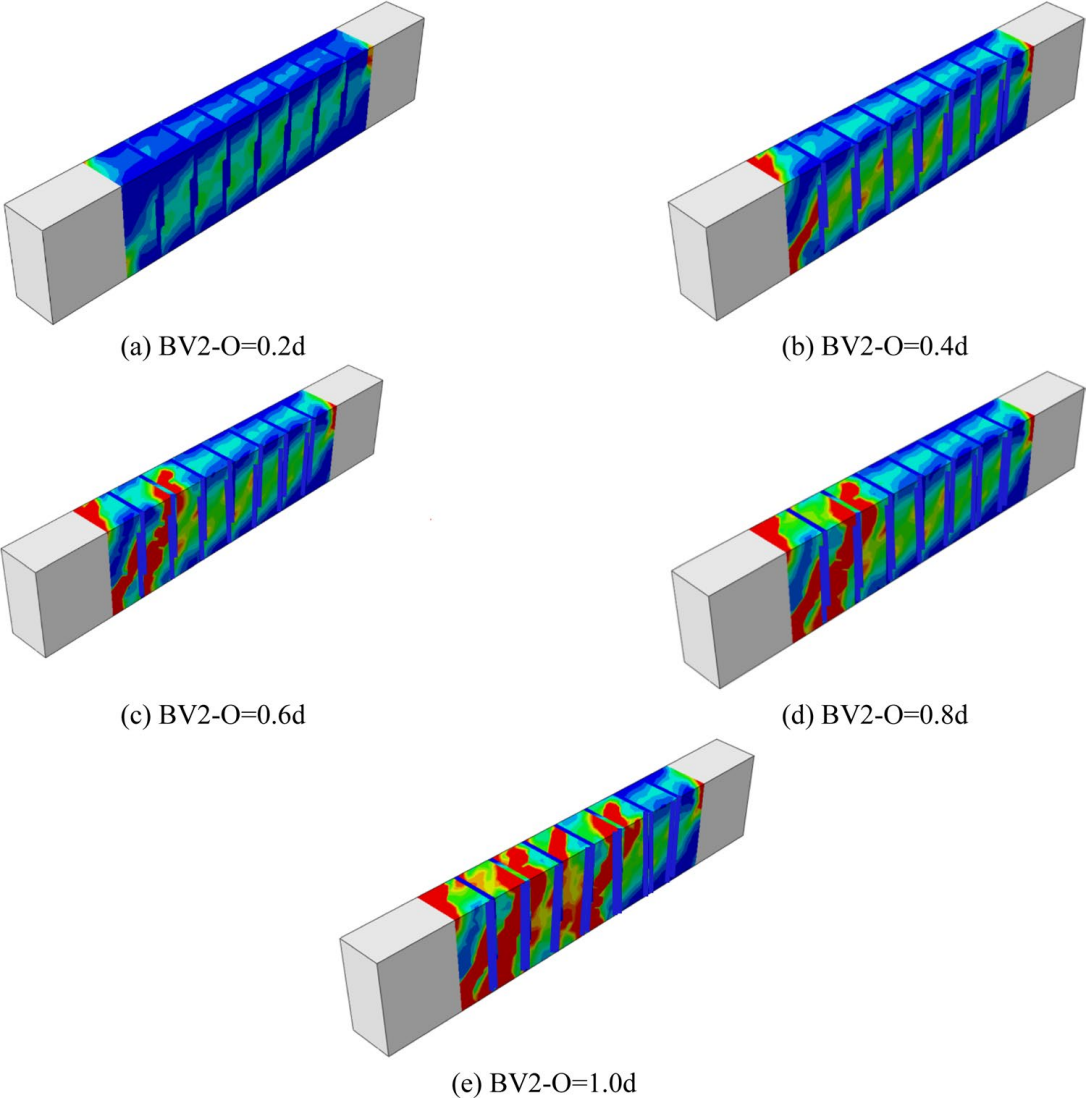
Numerical failure modes of beams with different overlap lengths (**a**) BV2-O = 0.2d, (**b**) BV2-O = 0.4d, (**c**) BV2-O = 0.6d, (**d**) BV2-O = 0.8d, (**e**) BV2-O = 1.0d.

Figure 16, shows the crack and collapse patterns resulting from the change in the overlap length of the reinforcement stirrups. The increase in the stirrup length led to an increase in the torsional load of the beams and led to the appearance of cracks and a delay in collapse. The images show the extent of crack spread on the faces of the beam as a result of the delay in reaching collapse, which means an increase in the plasticity and flexibility of the beam. However, the increase of 0.2d was not effective, but rather it appeared to increase the speed of collapse of the beam compared to the reference beam because the overlap length was not sufficient to bear the load compared to the cutting that occurred in the body of the beam. With the increase in the overlap length, the collapse pattern was similar to reality with an increase in the number of cracks.

#### Strengthening with combined NSM and EBR techniques

**Fig. 17:**
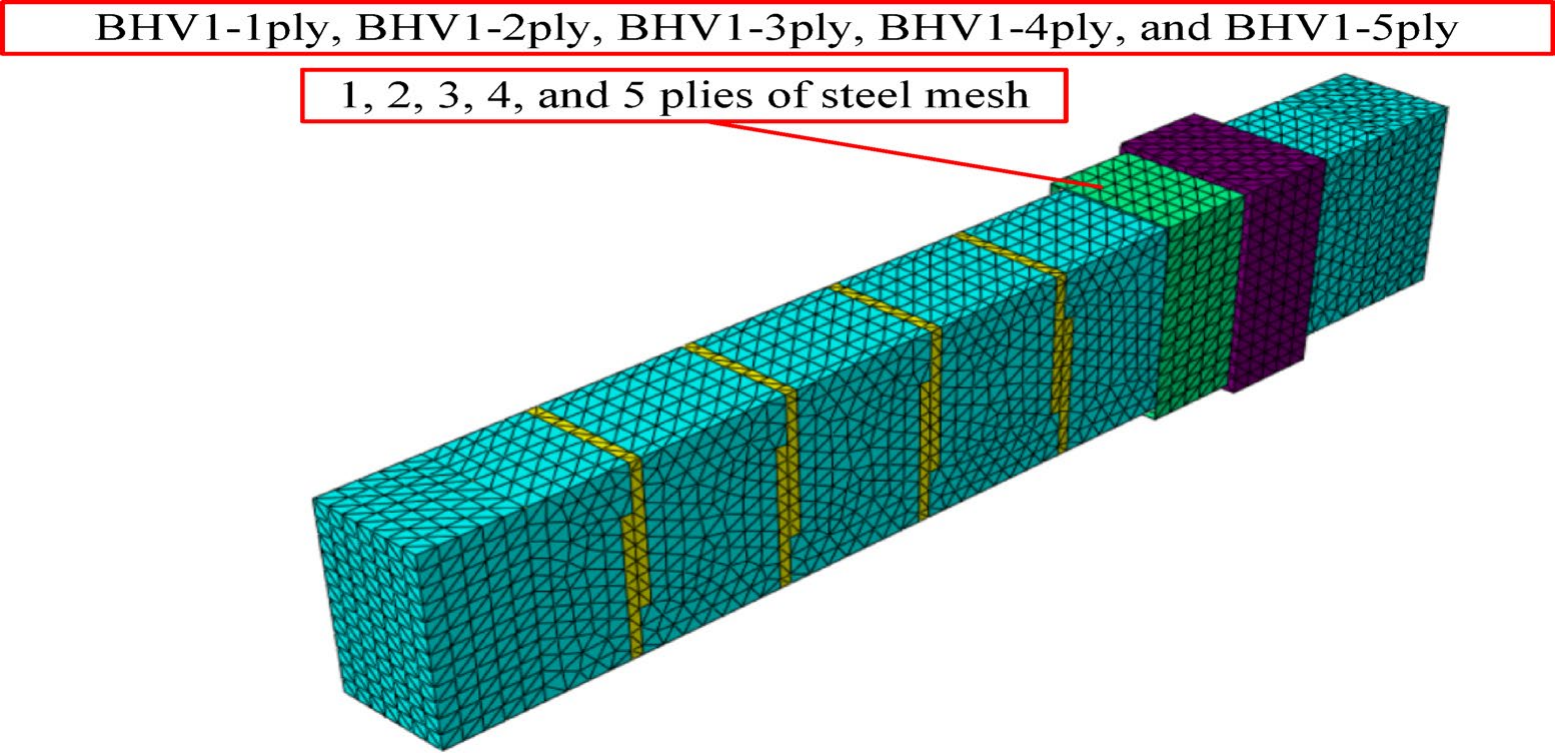
Beams of the parametric study with variable number of steel mesh layers.

A standard numerical study was conducted to evaluate the effectiveness of combined strengthening techniques for a reference BC beam, which demonstrated a reduction in torsional capacity due to the use of open internal stirrups. The beam was first strengthened using the BHV system, where H denotes the hybrid system, with external stirrups installed using the near-surface-mounting (NSM) technique with an overlap length of 0.5d (where d is the total depth of the beam). To further enhance the torsional strength, the external surface of the beam was subsequently re-erected using the external bracing (EBR) method, using a fabric-reinforced mortar (TRM) system with a steel mesh. Five configurations, consisting of one, two, three, four, and five layers of steel mesh, respectively, were analyzed and designated as BHV1—one layer, BHV2—two layers, BHV**3**—three layers, BHV4—four layers, and BHV5—five layers, as shown in Fig. 17.

##### Effect of strengthening with combined NSM and EBR techniques

**Fig. 18:**
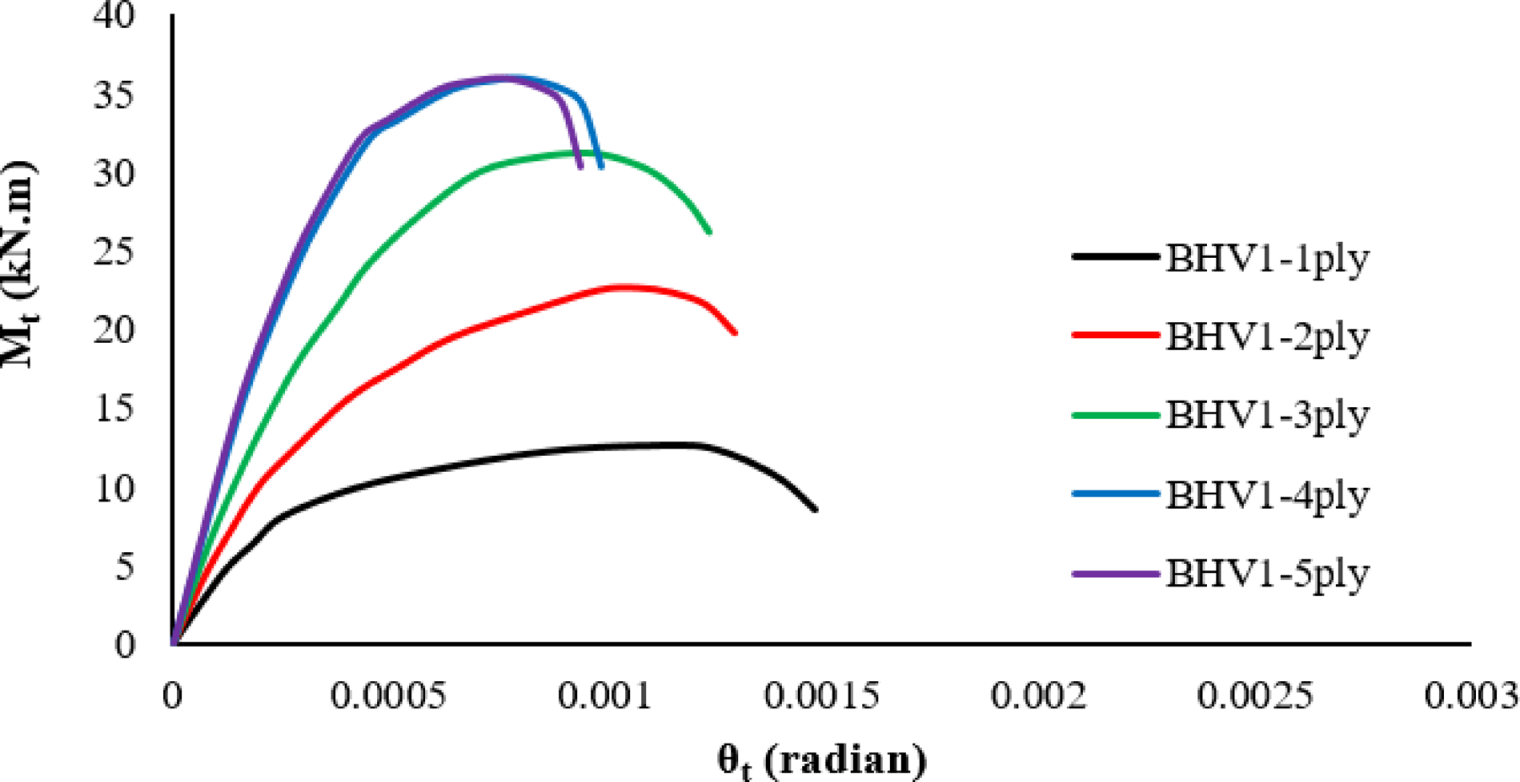
Twist angle–torsional moment curves of the tested beams.

**Fig. 19:**
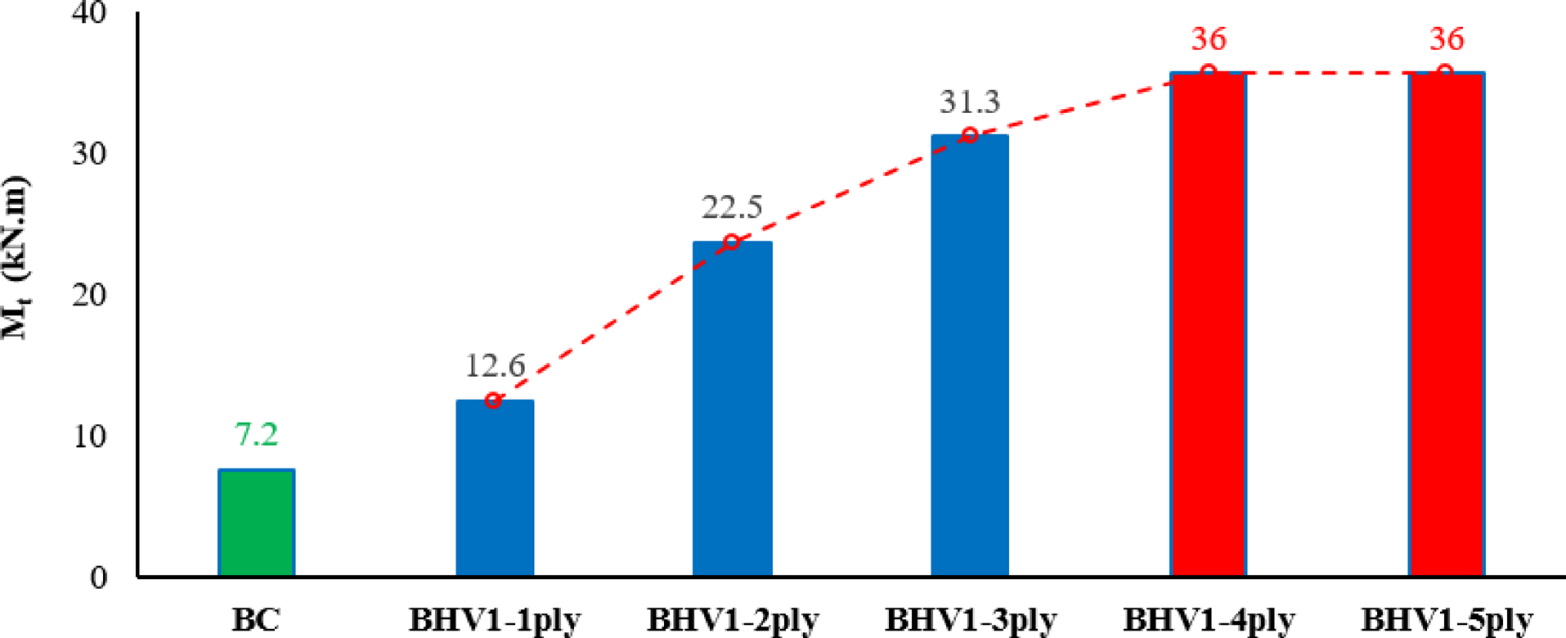
The effect of the number of steel mesh layers.

The numerical results shown in Table 6, show a significant improvement in torsional strength with the application of the combined strengthening systems. The reference BC beam carried a maximum torque of 7.2 kN/m, while the reinforced beams showed much higher torsional capacities, as the maximum torsional torque increased by 75%, 212%, 335%, 400%, and 400%, respectively, while the torsional angle increased by varying percentages of 71%, 43%, 43%, 14%, and 0%, respectively. These percentages demonstrate the effectiveness of using mesh layers in increasing the torsional moment and torsional angle, but using three mesh layers is the most appropriate number in terms of the effect of the increase, cost, and effort, as increasing to four and five layers was not an effective increase. The results also show that it increases the forms of brittle failure. The relationship between the torsional moment and the torsional angle was represented by a curve as shown in Figs. 18, 19.

**Table 6:** Results of changing the number of steel mesh layers.

Beams ID	Number of layers of steel mesh	M_t_ (kN.m)	Inc%	θ_t_ (radian)	Inc%
Bcon	0	7.2	0	0.0007	0
BHV1	1	12.6	1.75	0.0012	1.71
	2	22.5	3.12	0.001	1.43
	3	31.3	4.35	0.001	1.43
	4	36	5	0.0008	1.14
	5	36	5	0.0007	0

##### Numerical failure modes of beams

**Fig. 20:**
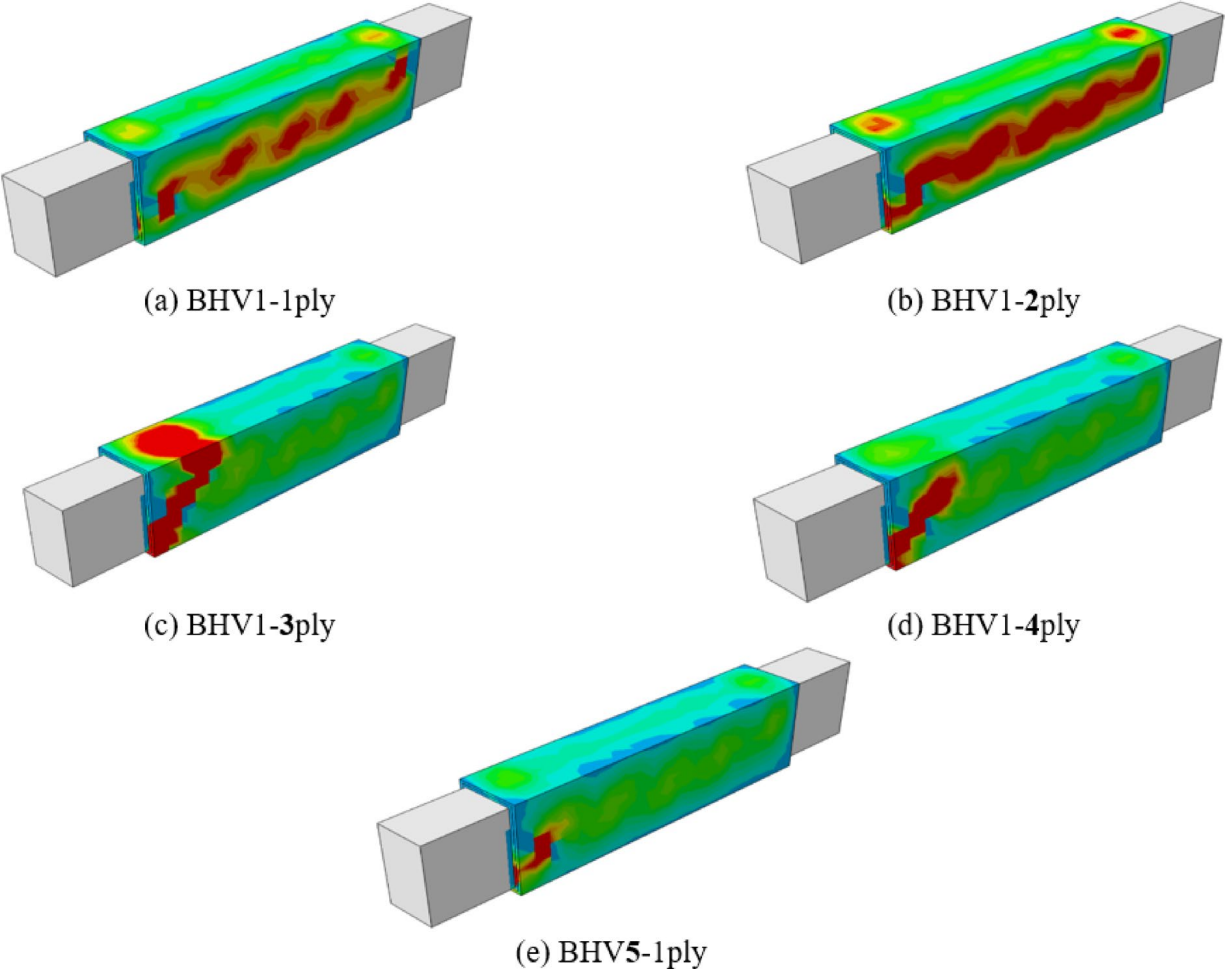
Numerical failure modes of beams with different numbers of steel mesh layers (**a**) BHV1-1ply, (**b**) BHV1-2ply, (**c**) BHV1-3ply, (**d**) BHV1-4ply, (**e**) BHV1-5ply.

Figure 20, shows the cracking and failure patterns resulting from the change in the number of layers of steel mesh. The increase in the number of layers of mesh led to a noticeable change in the pattern of spalling and collapse, as with an increase in the layer there was an increase in the spread and increase of cracks on the faces of the beams with an increase in the beam’s tolerance for a higher twisting moment until the number of layers reached three layers, a very clear difference appeared in the spread of cracks and their increase and the increase in the angle of torsion. Then after increasing the layers to four and five layers, the cracks appeared less and a brittle collapse occurred, and this is likely to have occurred. A sudden separation between the layers and the beam could lead to an almost sudden collapse due to the beam bearing a higher load and the lack of greater flexibility. Therefore, the best case for cracks and collapse was when using three layers, The optimal performance observed for three mesh layers can be attributed to an effective balance between confinement enhancement and deformation capacity. Up to three layers, stress redistribution and crack confinement improve torsional resistance and ductility. Beyond this limit, excessive stiffness reduces deformation capacity and promotes brittle behavior, limiting further performance gains.

### Inclined strengthening

**Fig. 21:**
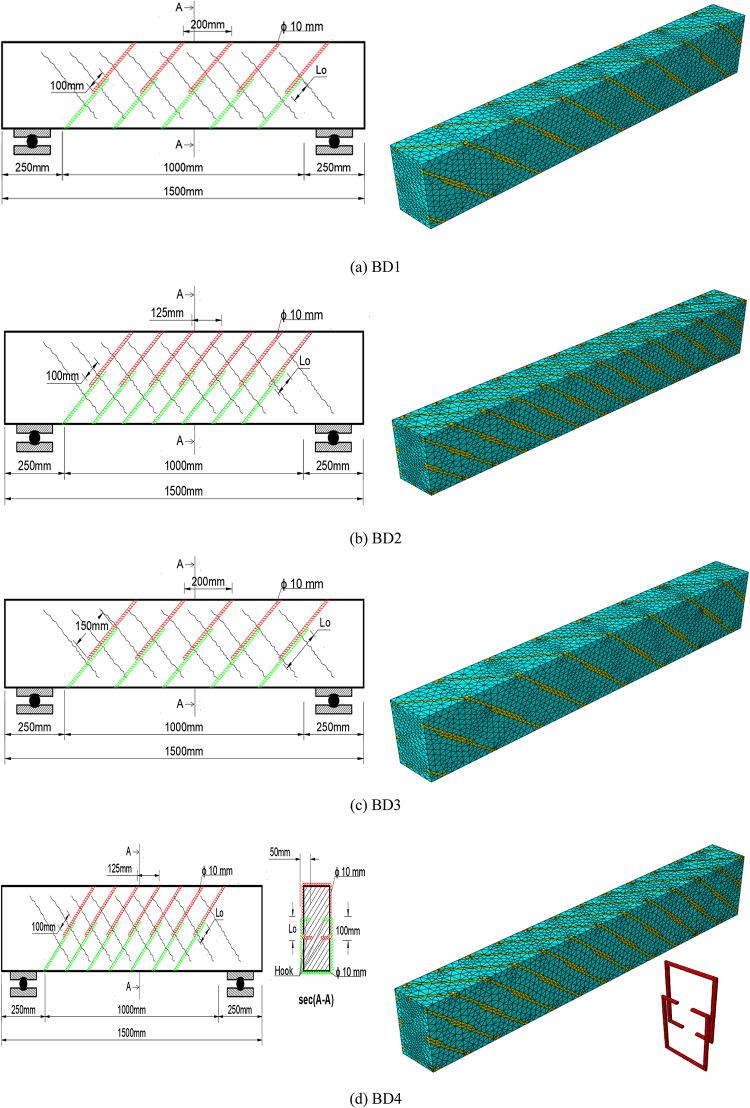
Numerical models of beams (**a**) BD1, (**b**) BD2, (**c**) BD3, (**d**) BD4.

Based on what is known, the cracks resulting from torsional loads are diagonal and, to a large extent, are inclined at an angle of 45 degrees on the outer surface of the beam and on all faces of the beam. Therefore, it was necessary to study the effect of external support in an inclined manner, so that it is perpendicular as much as possible to the cracks resulting from torsional loads. Therefore, four beams were selected, supported with the same inclined stirrups, but with several variables changed as shown in Table 7, Accordingly, the effect of the inclined external support stirrups was studied, with several factors also changed, such as the length of the overlap, the distances between the stirrups, and the use of a hook in the support stirrups inside the beam., as shown in Fig. 21.

**Table 7:** Details for tested beams.

No	Name	Strengthening					Notes
		Type			Overlap	Distance	
					Lo (mm)	S (mm)	
1	BD1	NSM	Steel bars Ø 10		100	200	U- Shap
2	BD2				100	125	
3	BD3				150	200	
4	BD4				100	125	hook

#### Effect of inclined strengthening

**Fig. 22:**
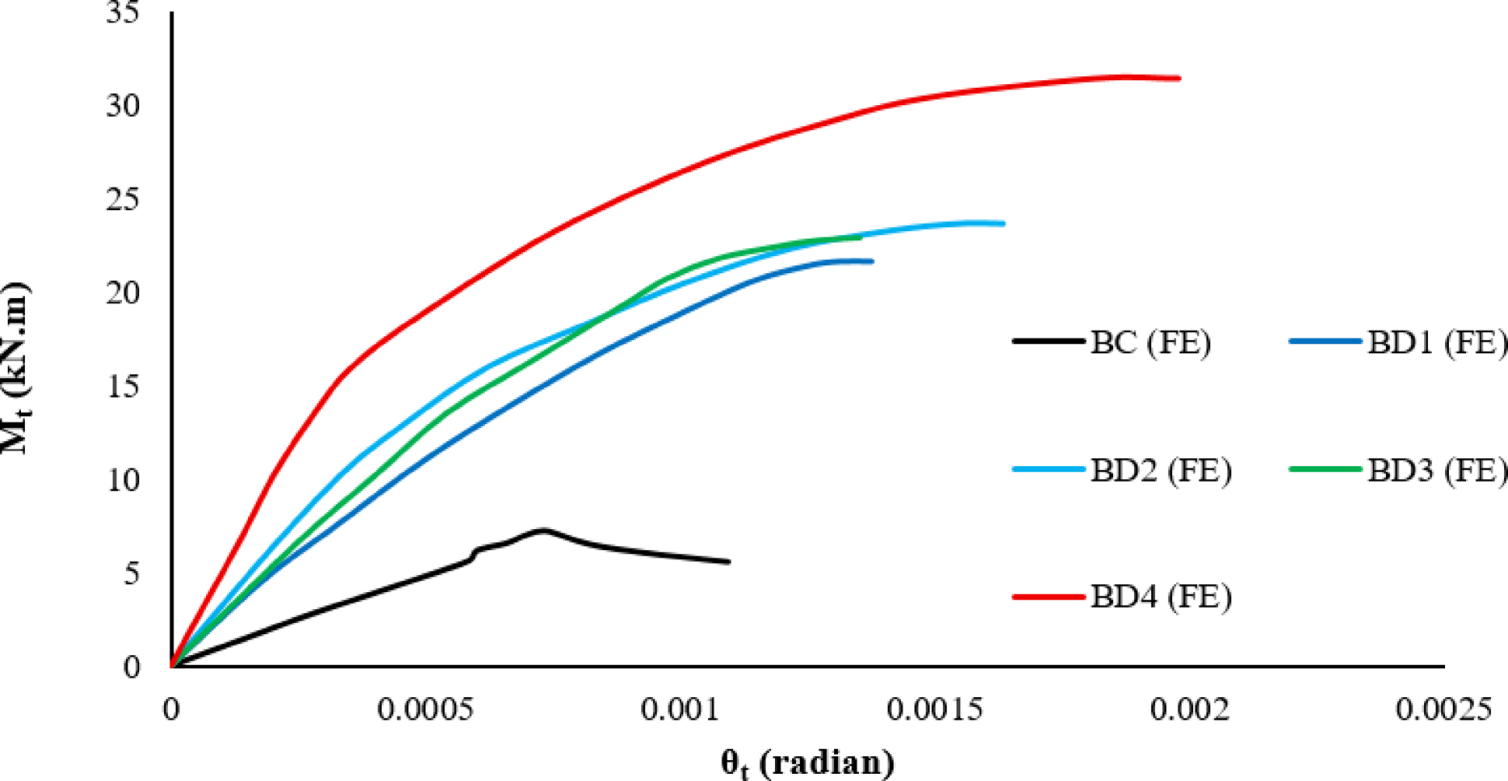
The relationship between the twisting moment and the twist angle for all beams.

**Fig. 23:**
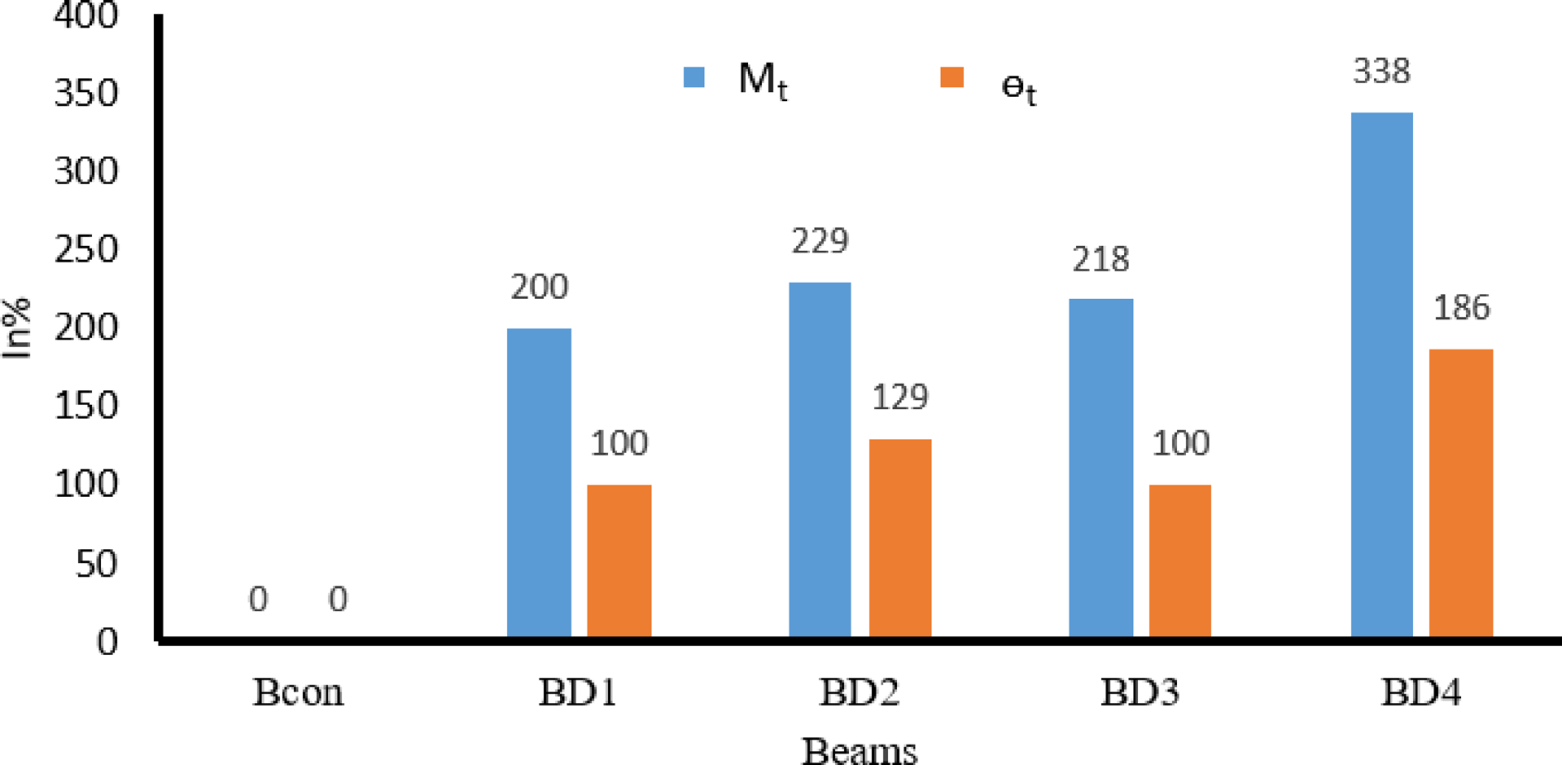
Comparison of all beams in twisting moment (M_t_) and the twist angle (ɵ_t_) for all beams.

The numerical analysis results showed a clear improvement in the torsional behavior of all the reinforced beams compared to the control beam Bcon, as shown in Table 8. The numerically optimized beams exhibited significant increases in both the ultimate torsional moment and the torsional angle as shown in Fig. 22, confirming the efficiency of the bracing system. Models BD1 and BD3 achieved moderate improvements, with increases ranging from 200 to 218% in torsional resistance and approximately 100% in the ultimate torsional angle. This indicates that the change in span length between 100 and 150 mm increased the torsional strength by 18% compared to the two beams, a relatively small difference, with limited improvement in deformability. Model BD2, however, demonstrated more balanced performance, achieving a higher 229% increase in resistance accompanied by a significant 129% increase in the torsional angle, indicating better stress transfer efficiency and a stronger interaction between the concrete and the bracing system. The BD4 model recorded the highest performance, achieving an exceptional 337% increase in torsional resistance and 186% in the angle of twist, reflecting the effectiveness of adding a hook inside the beam section. Overall, the numerical results confirm that all reinforcement methods improved torsional behavior to varying degrees, with the BD4 model demonstrating clear superiority in terms of strength and ductility as shown in Fig. 23.

**Table 8:** Results for tested beams.

Beams ID	M_t_ (kN.m)	In%	θ_t_ (radian)	In%
Bcon	7.2	0	0.0007	0
BD1	21.6	200	0.0014	100
BD2	23.7	229	0.0016	129
BD3	22.9	218	0.0014	100
BD4	31.5	338	0.002	186

#### Numerical failure modes of beams

**Fig. 24:**
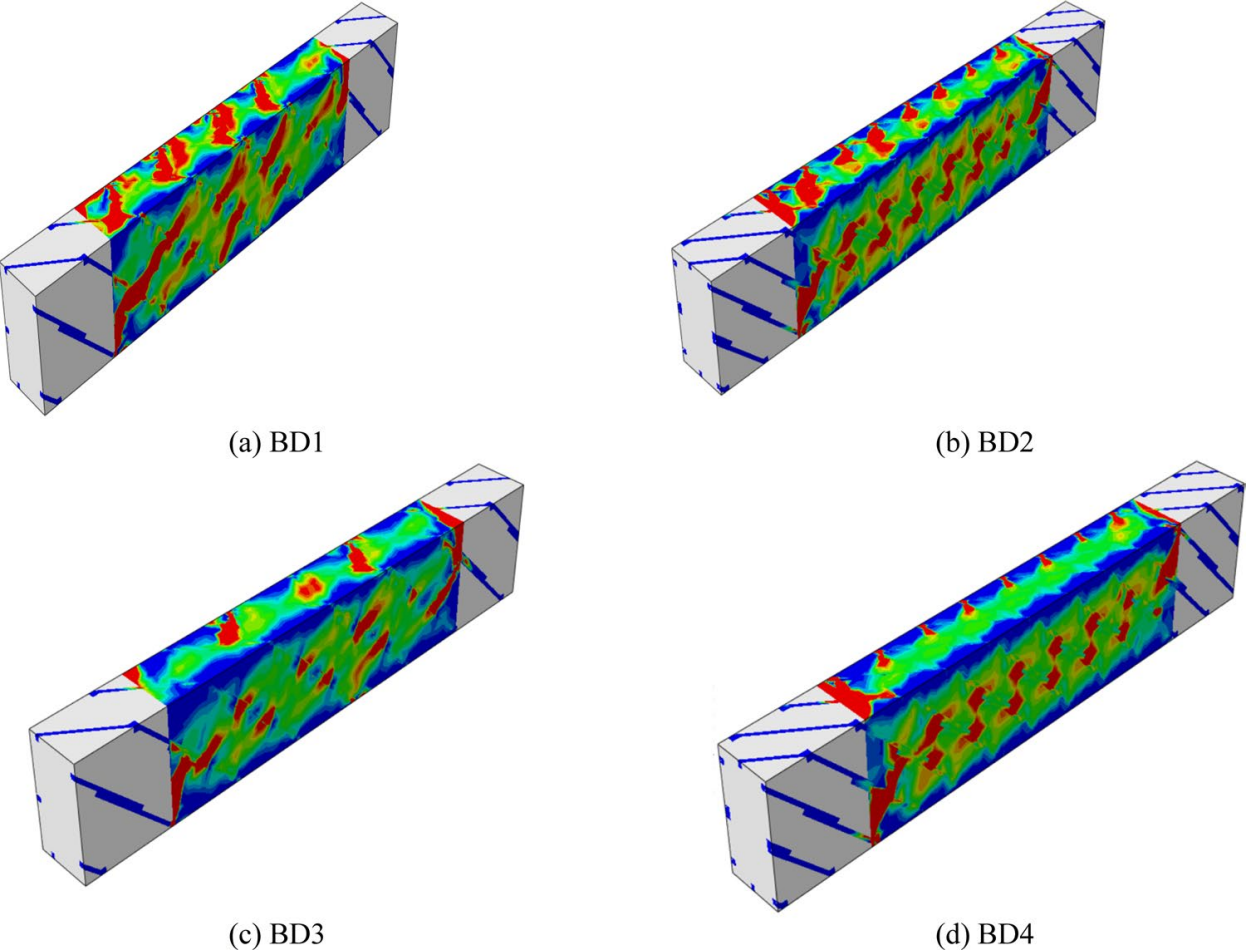
Numerical failure modes of beams with different numbers of steel mesh layers (**a**) BD1, (**b**) BD2, (**c**) BD3, (**d**) BD4.

The numerical results showed that all the reinforced beams experienced a diagonal failure pattern due to torsion, very close to a 45° angle as shown in Fig. 24. However, the severity of the deformation and the spread of the stress zones varied according to the bracing method used. BD1 exhibited relatively limited torsional cracking with stress concentration at the ends, indicating moderate strain-trapping effectiveness. BD2 showed a more uniform failure pattern with a clear spread of diagonal cracks along the beam, thanks to the bracing system’s emphasis on stirrup density. BD3 recorded a similar distribution but with a higher stress concentration at the two supports, reflecting variations in bracing effectiveness near the ends. In contrast, BD4 demonstrated the best numerical performance, with diagonal cracks spreading uniformly and extensively as stresses reached their peak before failure. This reflects the highest degree of confinement and bonding between the concrete and the bracing, and the highest capacity to withstand torsional deformation. These graded patterns confirm that the efficiency of the reinforcement system clearly increases from BD1 to BD4.

## Model limitations and design implications

Despite the high degree of agreement observed between numerical and experimental results, the developed finite element model is still subject to certain limitations. Assumptions of perfect bonding, material homogeneity, and the absence of construction defects may lead to a slight overestimation of stiffness and toughness. Therefore, numerical results should be interpreted as general indicators rather than precise predictions.

In practice, the results indicate that increasing the reinforcement intensity beyond certain thresholds such as overlap lengths exceeding 0.8d or more than three lattice layers reduces structural benefits and may promote brittle behavior. These results provide valuable guidance for selecting efficient and feasible torsional reinforcement configurations, rather than arbitrarily increasing reinforcement.

## Conclusions

The numerical study conducted in this research aimed to validate the experimental results and expand the scope of research into torsional strengthening techniques for reinforced concrete beams through a parametric study. The goal was to save time, effort, and cost, and to obtain reliable results. By developing a finite element (FE) model using Abaqus/CAE software, the complex structural response under pure torsion was accurately simulated. The comparison between the experimental and numerical results confirmed the reliability of the model and provided a solid foundation for further parametric studies. Based on the analyses and results presented, the following conclusions can be drawn:

1. The finite element (FE) model developed in Abaqus/CAE successfully reproduced the experimental behavior of reinforced concrete beams reinforced with NSM and hybrid systems under torsional stress.
2. The numerical predictions of cracking moment, maximum torque, and torsional angles showed very close agreement with the experimental results, with deviations not exceeding 5%.
3. The adopted constitutive models (damaged concrete ductility, interface bond zone, and ductile steel composition) demonstrated remarkable consistency in cracking patterns, stiffness degradation, and debonding phenomena.
4. A mesh sensitivity analysis confirmed that a 25 × 25 mm mesh size provides the best balance between computational efficiency, accuracy, and response speed.
5. The expansion angle (ψ) was identified as a highly sensitive parameter; a value of 37° achieved the best agreement with the experimental torque-torsion responses.
6. A comparison of the numerical and experimental crack patterns showed strong consistency in the crack initiation, propagation, and failure patterns.
7. The overlap length of the NSM external stirrups was found to be a key factor in increasing the beams’ ability to withstand higher torsional moments and greater torsional angles.
8. An overlap ratio of at least 0.4d between the NSM bracing branches is recommended for effective bracing.
9. Overlaps between 0.6d and 0.8d were determined to be optimal, balancing constructability and torsion resistance. They increased the bending moment by 110% to 138% and increased ductility by increasing the torsion angle by 14% to 86%.
10. Combining the NSM stirrups with externally bonded steel mesh layers significantly improved the torsional capacity.
11. Strength increased with increasing the number of layers up to three layers, increasing the bending moment by 112% and the bending angle by 43%. After 3 layers, the improvements became minimal, indicating a saturation limit and potential for brittle failure.
12. The finite element model proved to be a powerful and predictive tool, suitable for scaling up experimental results and conducting design-oriented parametric studies.
13. The results provide a solid basis for practical strengthening recommendations and future applications of NSM and hybrid modification systems for the torsional strengthening of reinforced concrete beams.
14. The inclined bracing system proved more efficient than the vertical system in increasing strength and ductility, with a 338% increase in torsional moment and a 186% increase in the angle of torsion.
15. The BD4 beam performed best among all models, achieving the highest increase in both torsional moment and angle of torsion.
16. Failure patterns showed a more uniform distribution of cracks in the inclined bracing beams, along with a greater capacity to accommodate deformation before failure.

## Data Availability

The data and materials supporting the findings of this study are available from the authors upon reasonable request.
